# TfmR, a novel TetR‐family transcriptional regulator, modulates the virulence of *Xanthomonas citri *in response to fatty acids

**DOI:** 10.1111/mpp.12786

**Published:** 2019-03-27

**Authors:** Doron Teper, Yanan Zhang, Nian Wang

**Affiliations:** ^1^ Citrus Research and Education Center, Department of Microbiology and Cell Science, Institute of Food and Agricultural Sciences University of Florida 700 Experiment Station Road Lake Alfred 33850 USA; ^2^ China-USA Citrus Huanglongbing Joint Laboratory (A joint laboratory of The University of Florida’s Institute of Food and Agricultural Sciences and Gannan Normal University), National Navel Orange Engineering Research Center Gannan Normal University Ganzhou 341000 Jiangxi China

**Keywords:** fatty acids, pathogenicity, TetR, type III secretion system, *Xanthomonas citri*

## Abstract

The type III secretion system (T3SS) is required for *Xanthomonas citri *subsp.* citri *(*Xcc*) virulence by translocating effectors into host cytoplasm to promote disease development. The T3SS is controlled by the master transcriptional regulators HrpG and HrpX. While the function of HrpG and HrpX are well characterized, their upstream regulation remains elusive. By using transposon mutagenesis, we identified XAC3052, a TetR‐family transcriptional regulator, which regulates T3SS gene expression. Deletion of XAC3052 caused significant reduction in the expression of T3SS and effector genes *in vitro *and *in planta*; as well as reduction of virulence in sweet orange (*Citrus sinensis*). Overexpression of *hrpG* restored the virulence of ∆XAC3052, suggesting that the loss of virulence is caused by reduction of T3SS gene expression. XAC3052 directly binds to the promoter region and represses the transcription of *fadE, mhpC* and *fadH* genes. FadE, MhpC and FadH are not involved in T3SS regulation, but involved in fatty acid catabolism. ∆XAC3052 displays altered fatty acid composition and retarded growth in environments limited in fatty acids. Exogenously supplemented long‐chain fatty acids activate the *fadE*/*mhpC* promoter and suppress T3SS promoters in wild‐type *Xac* but not in ∆XAC3052. Moreover, the binding of XAC3052 to its target promoter was disrupted by long‐chain fatty acids *in vitro*. Herein, XAC3052 is designated as TfmR (T3SS and Fatty acid Mechanism Regulator). This study identifies a novel regulator of fatty acid metabolism and suggests that fatty acids play an important role in the metabolic control of virulence in *Xcc*.

## Introduction

*Xanthomonas* is a large genus of plant pathogenic bacteria that cause diseases in almost all plant crops (Ryan *et al*., 2011). Most *Xanthomonas* pathogens utilize the type III secretion system to deliver effector proteins to manipulate host signalling to promote disease (Büttner and Bonas, 2010). Regulation of the type III secretion system (T3SS) and type III effector genes in *Xanthomonas* is dependent on the two master regulators HrpG and HrpX (Guo *et al*., 2011; Wengelnik and Bonas, 1996; Wengelnik *et al*., 1996). The OmpR family transcriptional regulator HrpG is induced during early infection (Potnis *et al*., 2015) and promotes the expression of the AraC‐type transcriptional activator *hrpX*, which in turn positively regulates the expression of the T3SS structural components and type III effector genes by binding to a conserved sequence motif (plant‐inducible promoter [PIP]) box) in the promoter regions of these genes (Koebnik *et al*., 2006; Kogenaru *et al*., 2012; Wengelnik and Bonas, 1996). HrpG and HrpX are subjected to complex regulation through transcription (Kametani‐Ikawa *et al*., 2011; Lu *et al*., 2011), transcript stability (Andrade *et al*., 2014), protein stability (Zhou *et al*., 2018) and post‐transcriptional modification (Li *et al*., 2014), and are under tight metabolic control (Rashid *et al*., 2016; Schulte, 1992). While many advances have been made in understanding the upstream regulation of HrpG and HrpX, the mechanism that enables the selective induction and activation of these proteins remains unclear.

TetR‐family regulators (TFRs) are a large family of bacterial transcriptional regulators (Ramos *et al*., 2005). Their regulatory function greatly varies and was found to be involved in multidrug resistance, catabolism, osmotic stress, quorum sensing, antibiotic production and host response (Ramos *et al*., 2005). TFRs typically function as transcriptional repressors that control the expression of their target genes in response to ligand molecules. All TFRs consist of an N‐terminal helix‐turn‐helix DNA binding domain and a C‐terminal domain that functions in dimerization and ligand interaction (Cuthbertson and Nodwell, 2013; Deng *et al*., 2013). In the absence of ligand, TFR homodimers bind to a palindromic operator sequence in their perspective target promoters and inhibit their transcription (Deng *et al*., 2013). In the presence of ligand, the ligand directly binds to the regulator and promotes a conformational change that releases the TFR from its target promoter and enables its transcription (Deng *et al*., 2013). The sequence or the operator, characteristic of the ligand and the number of target genes are unique to each TFR and vary greatly. In many cases, TFRs are located in small transcriptional units in divergent orientation to their target genes (Ramos *et al*., 2005).

In bacteria, long‐chain fatty acids are catabolized into acetyl‐CoA *via* the beta‐oxidation pathway and mediated by fatty acid degradation (Fad) enzymes (Fujita *et al*., 2007). The beta‐oxidation pathway is highly conserved but its regulation differs significantly and is controlled by TFRs in some bacteria: YsiA of *Bacillus subtilis* functions as a master regulator of Fad by controlling multiple beta‐oxidation coding genes in dependence of long‐chain fatty acyl‐coAs (Fujihashi *et al*., 2013; Matsuoka *et al*., 2007), PsrA of *Pseudomonas aeruginosa* controls the *fadB/A* operon and *fadE* in dependence of long‐chain fatty acids (Kang *et al*., 2008; Wells *et al*., 2017); and *Thermus* *thermophiles* FadR controls multiple beta‐oxidation related operons (Agari *et al*., 2011).

Most fatty acid related research in* Xanthomonas* is focused on the diffusible signal factor (DSF), a fatty acid derivative that acts as the main quorum sensing molecule (Guo *et al*., 2012; Li *et al*., 2019; Zhou *et al*., 2017a). Numerous studies were conducted regarding the metabolic control of DSF production and some have directly linked the production and sensing of DSF to fatty acid biosynthesis and beta‐oxidation related genes (Bi *et al*., 2014; Yu *et al*., 2016). However, very few studies have investigated the utilization of fatty acids as an energy source and regulation of the beta‐oxidation pathway itself and its relationship to virulence.

In this study, we identified the TFR protein TfmR as a novel virulence regulator in *Xanthomonas citri* subsp. *citri*, the causal agent of citrus canker (Vojnov *et al*., 2010). We found that TfmR acts as an indirect positive regulator of the T3SS and type III effector genes and a direct negative regulator of fatty acid catabolic genes, *fadE, mhpC* and *fadH*, in response to long‐chain fatty acids. We demonstrated that supplementing long‐chain fatty acids to T3SS inducing media suppresses the expression of virulence genes in dependence of TfmR. We provide novel insights into the regulation of fatty acid metabolism and the relationship between fatty acid homeostasis and virulence regulation in *Xanthomonas*.

## Results

### TfmR is required for pathogenicity of *Xcc*

We conducted random mutagenesis using EZ‐Tn5 transposon (Epicentre) to identify novel regulators of T3SS in *Xcc*. The Tn5 transposon was transformed into *Xcc* 306 strain that harbours a plasmid expressing β‐glucuronidase (GUS) under the control of the promoter region of the T3S translocon *hrpF* (Locus tag XAC0394). Clones that displayed reduction in GUS activity were tested for virulence in sweet orange and the Tn5 insertion site was determined in 16 clones that displayed reduced virulence (Fig. S1 and Table S1). Two independent Tn5 mutants identified during the EZ‐Tn5 transposon screen were mutated in TfmR (Locus tag XAC3052; Table S1, Fig. S2A and B) that encodes a TetR‐family transcriptional regulator (Fig. S2C). Homology search has identified that TfmR is conserved in all *Xanthomonas* spp. and its homologs are present in the majority of bacteria in the Xanthomonadales order but not in other bacteria outside of Xanthomonadales (Fig. S2C).

**Figure 1:**
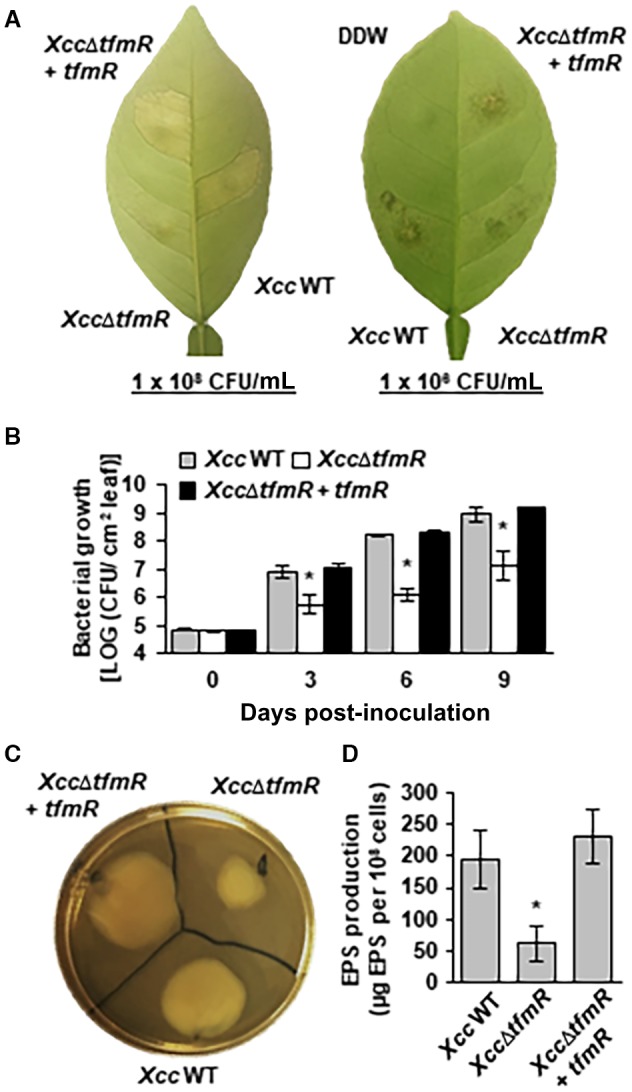
TfmR contributes to the virulence of *Xcc*. (A, B) sweet orange leaves were syringe‐infiltrated with suspensions (1 × 10^8^ CFU/mL for A, 1 × 10^6^ CFU/mL for A and B) of *Xcc* wild type (WT), *Xcc*∆*tfmR* and *Xcc*∆*tfmR* carrying complementation plasmid (+ *tfmR*). (A) Inoculated leaf was photographed at 5 (for 1 × 10^8^ CFU/mL) or 8 (for 1 × 10^6^ CFU/mL) days post‐inoculation. The experiments were repeated five times with similar results. (B) Bacterial growth *in planta*. Values are means ± SE of three independent biological repeats within one experiment. The experiment was repeated five times with similar results. (C) Mobility assay. Bacterial suspensions (1 × 10^8 ^CFU/mL) of the indicated strains were spotted (5 µl) on soft NA plates (0.4%). Three days later plates were photographed. (D) EPS production. The indicated strains were grown for 24 h in NB media +2% sucrose. Total EPS was quantified and standardized to the number of bacterial cells. Values are means ± SE of six independent biological repeats taken from three separate experiments (each containing two repeats). The experiment was repeated three times with similar results. (B, D) Asterisks indicate a significant difference (Student’s *t*‐test, *P*‐value < 0.05) relative to *Xcc* wild type.

To investigate whether the reduction in virulence in the TfmR Tn5 mutants is caused by disruption of *tfmR*, we generated a deletion mutant of *tfmR* (*Xcc*∆*tfmR*, Fig. S2A and D). Similar to the Tn5 mutants, sweet orange leaves infected with *Xcc*∆*tfmR* displayed less disease symptoms compared to that infected with the wild‐type *Xcc *(Fig. 1A). Additionally, *Xcc*∆*tfmR* bacterial population in sweet orange leaves was significantly reduced compared to the wild‐type *Xcc* at 3, 6 and 9 days post‐inoculation (dpi) (Fig. 1B). Reintroduction of *tfmR* gene into *Xcc*∆*tfmR* by plasmid restored canker symptoms and bacterial population to the wild‐type level (Fig. 1A and B). To further characterize the role of *tfmR *in bacterial pathogenesis we monitored multiple virulence associated traits. *Xcc*∆*tfmR* showed significant reduction in motility and production of exopolysaccharides (EPS) (Fig. 1C, D and S3A). On the other hand, *Xcc*∆*tfmR* did not affect hydrolase and catalase activity or resistance to exogenous SDS or hydrogen peroxide (Fig. S3).

### TfmR regulates T3SS and effector gene expression

**Figure 2:**
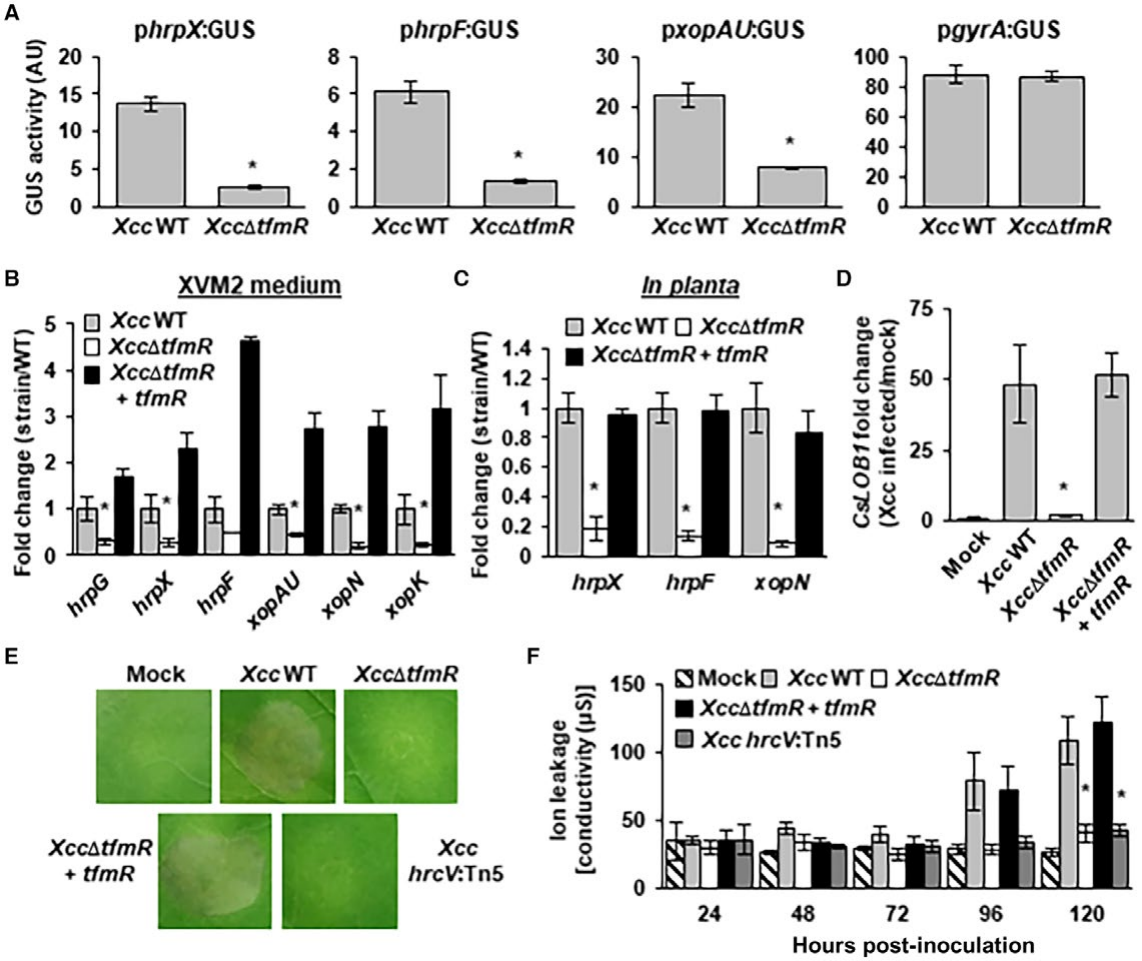
TfmR contributes to T3SS gene expression. (A) GUS activity (arbitrary units [AU]) was determined for *Xcc* wild type (WT) and *Xcc*∆*tfmR* harbouring the indicated GUS promoter fusions. *Xcc* strains were grown in XVM2 media for 24 h. Values are means ± SE of three independent biological repeats within one experiment. The experiments were repeated at least three times with similar results. (B) mRNA abundance of the indicated genes was quantified by qRT‐PCR in bacterial cultures after incubation in the XVM2 media for 24 h. *gyrA* mRNA abundance was used for normalization. Values are means ± SE of three independent biological repeats within one experiment. The experiment was repeated three times with similar results. (C, D) Sweet orange leaves were syringe‐infiltrated with suspensions (1 × 10^8^ CFU/mL) of the indicated strains. mRNA abundance of the indicated bacterial genes (C) and citrus *CsLOB1* (D) was quantified by qRT‐PCR 36 h post‐inoculation. Bacterial *gyrA* and citrus *GAPDH* mRNA abundance were used for normalization. Values are means ± SE of three independent biological repeats within one experiment. The experiment was repeated three times with similar results. (E, F). *N. benthamiana* leaves were syringe‐infiltrated with suspensions (5 × 10^8^ CFU/mL) of the indicated strains. (E) The inoculated area was photographed 6 days after infiltration. The experiment was repeated three times with similar results. (F) Electrolyte leakage in the inoculated areas at the indicated days post‐inoculation. Values are means ± SE of at least three independent biological repeats within one experiment. The experiment was repeated twice with similar results. (A, B, C, D, F) Asterisks indicate a significant difference (Student’s *t*‐test, *P*‐value < 0.05) relative to *Xcc* wild type.

The pathogenicity of *Xcc* is highly dependent on the delivery of T3Es via the T3SS (Yan and Wang, 2011; White *et al*., 2009). Since our transposon screen utilized *hrpF* promoter activity as an initial selection criteria, we hypothesized that TfmR regulates the expression of T3SS and T3Es. To test this hypothesis, *Xcc* wild type and *Xcc*∆*tfmR* were transformed with a series of reporter plasmids expressing GUS under the control of the promoter regions of the critical T3SS regulator *hrpX *(XAC1266), the T3E *xopAU* (XAC1171) and the T3SS translocon *hrpF*. To eliminate the possibility that *Xcc*∆*tfmR* may demonstrate reduction in transcription or in the stability of the GUS protein, *Xcc*∆*tfmR* was also introduced with a reporter plasmid expressing GUS under the control of the DNA gyrase subunit A (*gyrA, *XAC1631) promoter. When grown in a T3SS inducing medium, XVM2, *Xcc*∆*tfmR* displayed significant (*P‐*value < 0.05) reduction in GUS activity under the control of the promoters of *hrpX*, *xopAU* and *hrpF* but not that of *gyrA* (Fig. 2A). To examine the effect of TfmR on T3SS and T3E transcript accumulation, we monitored the expression of six T3SS genes (T3SS regulators *hrpX* and *hrpG*, T3SS translocon *hrpF* and T3Es *xopAU*, *xopN* and *xopK*) in XVM2 by quantitative reverse transcription polymerase chain reaction (qRT‐PCR). Similar to promoter activity assays the expression of the tested T3SS genes was reduced in *Xcc*∆*tfmR* compared to wild‐type *Xcc* and *Xcc*∆*tfmR* containing a complementation plasmid carrying *tfmR* (Fig. 2B).

The contribution of TfmR to expression of T3SS associated genes *in planta* was determined in sweet orange and non‐host plant *Nicotiana benthamiana*. In sweet orange we assessed the expression of three T3SS genes (*hrpX*, *hrpF *and *xopN*) and the expression of citrus canker susceptibility gene *CsLOB1* (Hu *et al*., 2014) at 36 h after infection using qRT‐PCR. Expression of *CsLOB1* was reported to be directly induced by *Xcc* T3E PthA4, and therefore increased mRNA accumulation of this transcript can serve as a marker for detecting active translocation of PthA4 through the T3SS (Hu *et al*., 2014). The expression of the tested T3SS genes and *CsLOB1* was significantly (*P*‐value < 0.05) reduced in leaves infected with *Xcc*∆*tfmR* compared to *Xcc* wild type and *Xcc*∆*tfmR* complemented strain (Fig. 2C and D), indicating the TfmR contributes to T3SS gene expression and effector translocation *in planta*. We previously reported that T3SS gene expression is required for *Xcc* induction of hypersensitive response (HR) in *N. benthamiana *(Zhou *et al*., 2015, 2017b)*. Xcc* cells were infiltrated into leaves of *N. benthamiana* and HR‐like cell death was monitored visually and by quantification of ion leakage. Leaves infected with *Xcc*∆*tfmR* or *Xcc* *hrcV*:Tn5, a mutant in one of the structural genes of type III secretion system apparatus (Fenselau *et al*., 1992), developed weakened and delayed cell death compared to *Xcc* wild type and *Xcc*∆*tfmR* complemented strain (Fig. 2E and F), thus further establishing the contribution of TfmR to T3SS translocation *in planta*.

**Figure 3:**
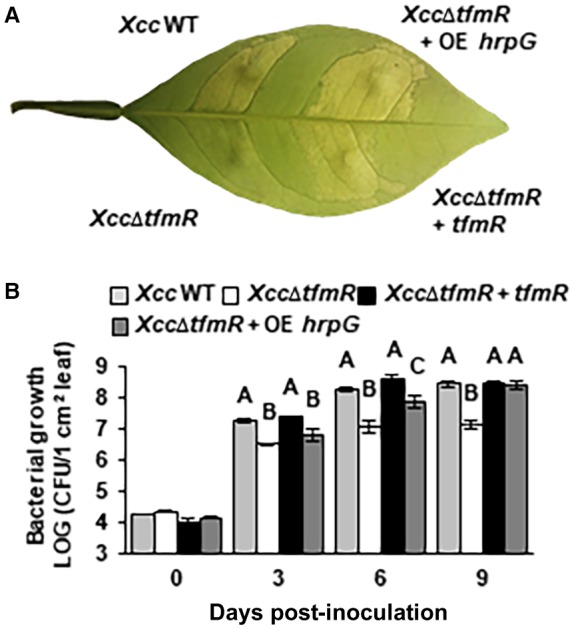
Complementation of *Xcc*∆*tfmR* by overexpression of HrpG. Sweet orange leaves were syringe‐infiltrated with suspensions (1 × 10^8^ CFU/mL for A, 1 × 10^6^ CFU/mL for B) of *Xcc* wild type (WT), *Xcc*∆*tfmR*, *Xcc*∆*tfmR *+ *tmfR*, and *Xcc*∆*tfmR* carrying a plasmid expressing *hrpG* under the control of a *lac* promoter (+ OE *hrpG*). (A) Inoculated leaf was photographed at 5 days post‐inoculation. The experiment was repeated three times with similar results. (B) Bacterial growth *in planta*. Values are means ± SE (*n* = 3). Values are means ± SE of three independent biological repeats within one experiment. The experiment was repeated three times with similar results. Letters denote significant differences based on analysis of variance (anova) and comparisons for all pairs using Student’s *t*‐test (*P*‐value < 0.05).

The reduction of virulence of *Xcc*∆*tfmR* in citrus may result from the reduction in the expression of T3SS and effector genes *in planta* or through other mechanisms. To examine if T3SS expression is responsible for this phenotype *in planta*, we introduced to *Xcc*∆*tfmR* a plasmid, which constitutively expressed the T3SS master regulator HrpG under the *lac* promoter. We then tested whether the constitutive expression of HrpG can bypass the requirement of *tfmR* for bacterial growth and symptom development. Constitutive expression of HrpG in *Xcc*∆*tfmR* restored symptom development and bacterial growth *in planta* (Fig. 3A and B). This indicates that the virulence reduction in *Xcc*∆*tfmR* possibly results from the decreased expression of T3SS genes.

### TfmR does not bind to the promoter of *hrpG* and *hrpX* directly

TetR proteins have been known to regulate target genes by binding to their promoter regions with their characteristic helix‐turn‐helix (HTH) DNA binding motif (Ramos *et al*., 2005). We therefore hypothesized that TfmR might control the T3SS and T3Es by directly controlling the expression of the T3SS master regulators *hrpG* or *hrpX*. We tested direct interaction between TfmR and the promoter regions of *hrpX* and *hrpG* *in vitro* and *in vivo* by electrophoretic mobility shift assay (EMSA) and Chromatin Immunoprecipitation‐Quantitative Polymerase Chain Reaction (ChIP‐qPCR) (Kim and Dekker, 2018). For EMSA analysis the 467 bp, 699 bp and 189 bp sequences upstream of the start codons of *hrpX*, *hrpG *and *gyrA* (DNA gyrase gene, used as a negative control), respectively, were used as probes and incubated with purified TfmR protein fused to Glutathione S‐transferase (GST) tag. No interaction was observed between GST‐TfmR and the *hrpX*, *hrpG* or the *gyrA* promoters (Fig. S4A). ChIP‐qPCR analysis was conducted by purifying TfmR fused to hemagglutinin (HA) from *Xcc* and quantifying the relative abundance of the intragenic region between *hrpX* and *hrpG *in the purified samples by qRT‐PCR. Similarly to the *in vitro* analysis, we did not observe direct interaction between TfmR and the *hrpG/hrpX* promoters *in vivo* (Fig. S4B). This indicates that the effect of TfmR on the transcription of T3SS and T3E genes is likely to be indirect.

### TfmR is a transcriptional repressor of the *mhpC/fadE* operon

**Figure 4:**
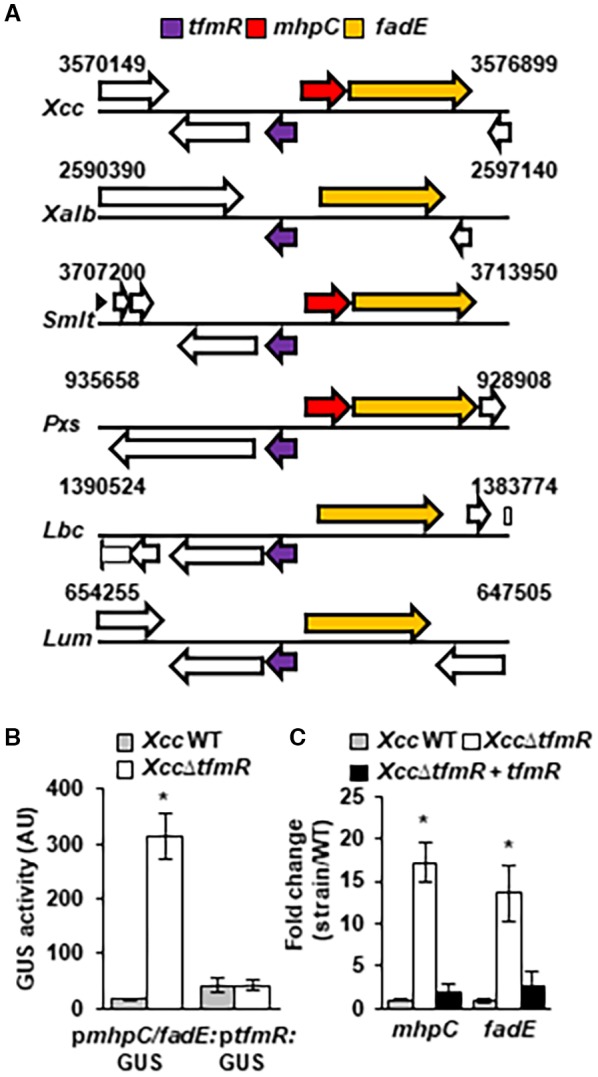
TfmR represses the expression of *mhpC *and* fadE*. (A) Physical map of the surrounding regions of the homologs of *tfmR* in *X. citri* sp. *citri* 306 (*Xcc*), *X. albilineans* GPE PC73R (*Xalb*), *Stenotrophomonas maltophilia* K279a (*Smlt*), *Pseudoxanthomonas spadix* BD‐a59 (*Pxs*), *Lysobacter capsici* 55 (*Lbc*) and *Luteimonas* sp. JM171 (*Lum*). ORFs encoding the homologs of *tfmR* are represented in purple, ORFs encoding *mhpC* are represented in red and ORFs encoding *fadE* are represented in orange. (B) Quantification of GUS in *Xcc* wild type (WT) or *Xcc*∆*tfmR* harbouring plasmids containing GUS promoter fusions of the *mhpC/fadE* operon and *tfmR*. *Xcc* strains were grown in NB media for 12 h. Values are means ± SE of three independent biological repeats within one experiment. The experiments were repeated three times with similar results. (C) mRNA abundance was quantified by qRT‐PCR in bacterial cultures after incubation in NB media for 12 h. *gyrA* mRNA abundance was used for normalization. Values are means ± SE of three independent biological repeats within one experiment. The experiment was repeated three times with similar results. (B, C) Asterisks indicate a significant difference (Student’s *t*‐test, *P*‐value < 0.05) relative to *Xcc* wild type.

The majority of transcriptional regulators of the TetR‐family are found in a divergent orientation to a neighbouring gene, which is directly regulated by them and many also regulate their own transcription (Cuthbertson and Nodwell, 2013). Upon examination of the available genome sequence deposits of bacteria from the Xanthomonadale order in the Integrated Microbial Genomes and Microbiomes database (https://img.jgi.doe.gov/), we identified that *tfmR* and its homologs in different *Xanthomonadales *are found in an opposite orientation to an operon encoding *fadE* (XAC3054 in *Xcc*) and *mhpC/bioH* family methyl ester carboxylesterase (XAC3053 in *Xcc*, absent in *X. albilineans*, *Lysobacter* and *Luteimonas*) (Fig. 4A). Therefore, we hypothesized that this operon is under the control of TfmR. To test this, a GUS reporter was placed under the control of the promoter region of XAC3053/XAC3054 (p*mhpC/ade*) and TfmR (p*tfmR*) (Fig. 4B), and the GUS activity from each promoter was tested in *Xcc* wild type and *Xcc*∆*tfmR*. No difference was observed in the promoter activity of p*tfmR*, whereas the promoter activity of p*mhpC/ade* was 50‐ to 100‐fold higher in *Xcc*∆*tfmR* compared to the wild type (Fig. 4B). Up‐regulation of both *mhpC *and *ade* was also observed in *Xcc*∆*tfmR* by qRT‐PCR compared to *Xcc* wild type and the *Xcc*∆*tfmR* complemented strain (Fig. 4C).

### TfmR directly binds to the promoter of the *mhpC/fadE *operon

**Figure 5:**
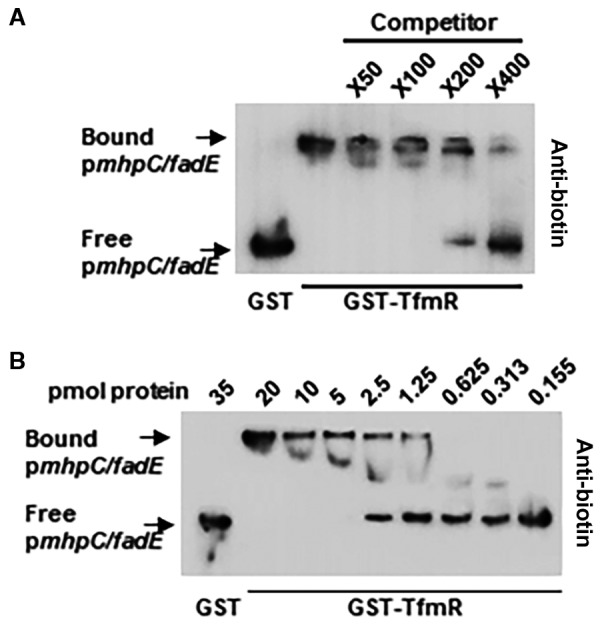
TfmR interacts with the promoter region of *mhpC/fadE*. Electrophoretic mobility shift assays (EMSA) was used to determine the binding of biotin labelled DNA by GST‐TfmR. (A) 20 pmol of GST or GST‐TfmR was incubated with 1 ng of biotin labelled p*mhpC/fadE *and the indicated fold increase of unlabelled p*mhpC/fadE *DNA as a competitor. The experiment was repeated three times with similar results. (B) Binding affinity assay of 1 ng of biotin labelled p*mhpC/fadE *with the indicated amount of GST‐TfmR or GST. The experiment was repeated three times with similar results.

We next tested the direct interaction between TfmR and the promoter regions of *mhpC/ade* (p*mhpC/ade* ) using EMSA and ChIP‐qPCR. Significant shift was observed when GST‐TfmR was incubated with p*mhpC/ade* compared to incubation with GST alone (Figs 5A and S4A). This shift was disrupted by the addition of a competitor, i.e. unlabelled p*mhpC/ade* DNA, in a dosage dependent manner (Fig. 5A). Direct binding was also observed *in vivo* using ChIP‐qPCR analysis that displayed enrichment of p*mhpC/ade* in the TfmR‐HA purified samples (Fig. S4B). We characterized the affinity of GST‐TfmR to p*mhpC/ade* DNA probe using decreasing protein concentrations and the KD value was determined to be 86.97 ± 21.83 nM (Fig. 5B). This indicates that the effect of TfmR on the transcription of its divergent neighbouring genes is based on direct interaction with the corresponding promoter sequences.

We next examined if the virulence reduction observed in *Xcc∆tfmR* is dependent on the *pmhpC/ade* operon. To test this we deleted the entire genomic region containing *tfmR*, *mhpC *and* ade* and assessed whether the deletion of *mhpC *and* ade* restores the virulence phenotype of *Xcc*∆*tfmR*. Deletion of *mhpC/ade* enabled the bacteria to grow slightly better in sweet orange compared to *Xcc*∆*tfmR *but did not complement the virulence reduction phenotype of *Xcc*∆*tfmR* and leaves inoculated with the triple mutant displayed slightly weaker disease symptoms than *Xcc*∆*tfmR* (Fig. S5A and B). Additionally, we overexpressed *mhpC/ade* through a plasmid under control of the *lac* promoter in the *Xcc* wild type. Overexpression of *mhpC/ade* did not significantly alter the virulence of *Xcc *(Fig. S5C and D).

### Fatty acids mediate the repression of the *mhpC/fadE *operon by TfmR

**Figure 6:**
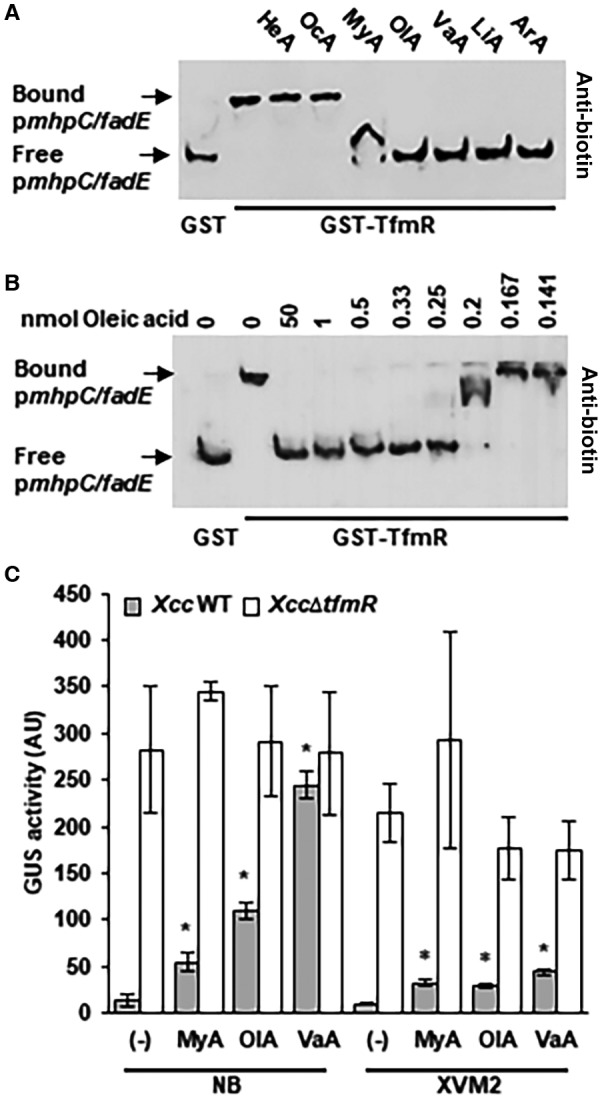
Long‐chain fatty acids modulate the DNA binding affinity of TfmR. (A, B) 20 pmol of GST‐TfmR was incubated for 15 min with 1 ng of biotin labelled p*mhpC/fadE *and binding was determined by EMSA. (A) Samples were incubated with 0.02% of the indicated fatty acids. The experiment was repeated three times with similar results. (B) Binding of p*mhpC/fadE *was tested in the indicated concentrations of oleic acid. The experiment was repeated four times with similar results. (C) GUS activity was determined for *Xcc* wild type (WT) and *Xcc*∆*tfmR* harbouring the p*mhpC/fadE *GUS promoter fusion. Bacteria were grown for 12 h in NB media or 24 h in XVM2 media supplemented with the indicated fatty acids. Values are means ± SE of three independent biological repeats within one experiment. The experiments were repeated three times with similar results. Asterisks indicate a significant difference (Student’s *t‐*test, *P*‐value < 0.05) relative to *Xcc* grown in NB media, which was not supplemented with fatty acids.

Several TFRs were reported to directly regulate fatty acid catabolism in response to long‐chain fatty acids in different bacteria (Fujita *et al*., 2007; Kang *et al*., 2008) (Agari *et al*., 2011). FadE and MhpC/BioH are directly controlled by TfmR in *Xcc*, and play an essential role in the catabolism of fatty acids (DiRusso *et al*., 1999; Ruslan *et al*., 2003) (Kadisch *et al*., 2017). Therefore, we hypothesized that TfmR responds to fatty acids in its regulation. We monitored the effect of multiple fatty acids on DNA binding by TfmR using EMSA (Fig. 6A). Addition of 0.02% of the short‐chain fatty acids, hexanoic (C6:0) acid and octaneic acid (C8:0) did not affect DNA binding, whereas the addition of 0.02% of multiple long‐chain fatty acids (*i.e,* myristic acid [C14:0], vaccenic acid [C18:1 cis‐7], oleic acid [C18:1 cis‐9], linoleic acid [18:2 cis‐9, 12] and arachidonic acid [cis‐5,8,11,14]) inhibited the binding of TfmR to its target promoter (Fig. 6A). We also demonstrated that the inhibition by oleic acid is concentration dependent (Fig. 6B).

The effect of fatty acids on the promoter activity of *mhpC/fadE *was tested in *Xcc* wild type and *Xcc*∆*tfmR *in nutrient broth (NB) and XVM2 media. Addition of 0.1% myristic acid, oleic acid and vaccenic acid to *Xcc* culture media significantly (*P*‐value < 0.05) induced the *mhpC/fadE *promoter in *Xcc* wild type by 5‐ to 30‐fold in NB and by 3‐ to 5‐fold in XVM2 (Fig. 6C). Exogenous supplementation of same fatty acids to *Xcc*∆*tfmR* cultures grown in either NB or XVM2 media did not affect the expression of the *mhpC*/f*adE* promoter (Fig. 6C), indicating that long‐chain fatty acids positively regulate the *mhpC*/*fadE *promoter probably by nullifying the repressing activity of TfmR.

### TfmR is important for fatty acid metabolism

**Figure 7:**
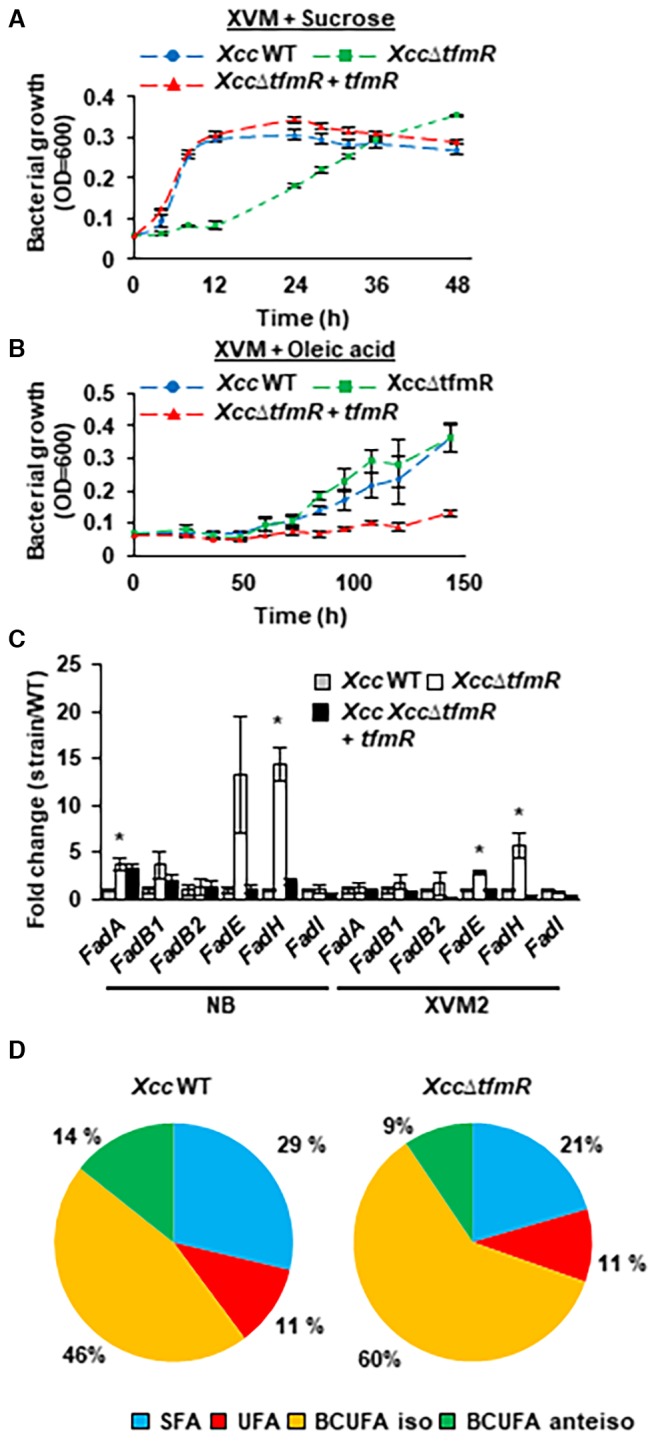
TfmR regulates fatty acid metabolism. (A, B) Growth of *Xcc* wild type (WT), *Xcc*∆*tfmR*, *Xcc*∆*tfmR* + *tfmR *in the XVM media containing 10 mM sucrose (A) or 0.1% oleic acid (B). Values are means ± SE of three (for XVM + sucrose) or four (for XVM + oleic acid) independent biological repeats within one experiment. The experiments were repeated twice (for XVM + sucrose) or three (for XVM + oleic acid) times with similar results. (C) mRNA abundance of the indicated genes was quantified in bacterial cultures grown in NB or XVM2 media by qRT‐PCR. *gyrA* mRNA abundance was used for normalization. Values are means ± SE of three independent biological repeats within one experiment. The experiments were repeated three times with similar results. Asterisks indicate a significant difference (Student’s *t*‐test, *P‐*value < 0.05) relative to *Xcc* wild type. (D) Fatty acid profiles of *Xcc* wild type (WT) and *Xcc*∆*tfmR*. The pie chart represents the average relative abundance in three independent bacterial samples. Detailed information showing the abundance of each fatty acid is found in Table S2.

The long‐chain fatty acid dependent regulation of *fadE* and *mhpC* by TfmR led us to speculate that deletion of this regulator might alter the fatty acid metabolic profile and regulation in *Xcc*. First, we examined if* Xcc*∆*tfmR* utilizes fatty acid and non‐fatty acid based carbon sources differently from the wild type. To this end *Xcc *wild type, *Xcc*∆*tfmR* and *Xcc*∆*tfmR* + *tmfR* were monitored for growth in XVM media (identical to XVM2 media minus fructose and sucrose) containing either 10 mM sucrose or 0.1% oleic acid. *Xcc*∆*tfmR* displayed growth retardation in XVM media supplemented with sucrose compared to the wild type and complemented strain but displayed similar growth kinetics to the wild type when the media were supplemented with oleic acid (Fig. 7A and B). *Xcc*∆*tfmR* also display reduced growth in XVM2 media in a similar manner to XVM + Sucrose (data not shown). Interestingly, the complemented strain, which expresses TfmR under the constitutive *lac* promoter, grew much slower than the wild type or the mutant strain in the presence of oleic acid (Fig. 7B). We further inspected how *Xcc*∆*tfmR *grows in rich media with or without exogenous supplementation of fatty acids. Bacteria were monitored for growth in NB, an undefined rich media or NB supplemented with 0.1% oleic acid. The growth rate of *Xcc*∆*tfmR *and *Xcc*∆*tfmR *complemented strain was slightly reduced compared to *Xcc* wild type in NB (Fig. S6A). In addition, *Xcc*∆*tfmR *cultures entered stationary phase upon reaching a lower population level than the wild type (Fig. S6A). The growth rate of both strains was similar to *Xcc* wild type when media was supplemented with oleic acid; however, *Xcc*∆*tfmR* entered stationary growth phase in a lower population compared to the wild type as well (Fig. S6B).

To further assess the effect of TfmR on fatty acid metabolism, we mapped key enzymes in the beta‐oxidation pathways in *Xcc* (*i.e*. *fadH *[XAC1010], *fadI *[XAC0213], *fadA *[XAC2012]) and two copies of *fadB *(*fadB1 *[XAC2013], in an operon with *fadA* and XAC2014, and *fadB2* [XAC1318]) and monitored the expression of these genes in *Xcc* wild type and *Xcc*∆*tfmR* using qRT‐PCR and GUS promoter fusions in NB and XVM2 media. In a similar manner to the expression pattern of *fadE*, *fadH* exhibited significantly (*P*‐value < 0.05) elevated mRNA transcript accumulation and promoter activity in *Xcc*∆*tfmR* compared to the *Xcc* wild type (Figs 7C and S6C). Other beta‐oxidation genes showed altered expression in *Xcc*∆*tfmR* compared to the wild type as well: media specific activation of the *fadI* promoter was observed in *Xcc*∆*tfmR *grown in XVM2 (Fig. S6C) while the mRNA transcript of *fadA* was consistently elevated in *Xcc*∆*tfmR *grown in NB media (Fig. 7C). Direct interaction of TfmR with the promoter regions of beta‐oxidation genes was determined *in vitro* and *in vivo* by EMSA and ChIP‐qPCR, respectively. TfmR was able to directly bind to the *fadH* promoter *in vitro* and *in vivo, *whereas binding to the promoter of XAC2014*/fadB1/fadA* was only observed *in vitro *(Figs S4B, S6D and S6E). Those results suggest that TfmR directly regulates multiple components of the fatty acid catabolism pathway.

MhpC/BioH was shown to play a role in the processing of fatty methyl‐esters (Kadisch *et al*., 2017; Ruslan *et al*., 2003) while FadE and FadH play an essential role in the beta‐oxidation cycle (Campbell and Cronan, 2002; He *et al*., 1997). The constitutive expression of these enzymes can potentially cause metabolic imbalance and alter the fatty acid profile of *Xcc*. We determined the fatty acid profile of *Xcc* wild type and *Xcc*∆*tfmR *by gas‐chromatography (GC) and found significant (*P*‐value < 0.05) differences in the abundance of most fatty acids and in the ratio of saturated and non‐saturated fatty acids as a whole. The percentage of saturated fatty acids within total fatty acids was higher in *Xcc* wild type compared to *Xcc*∆*tfmR*, while the percentage of non‐saturated fatty acids was higher in *Xcc*∆*tfmR* compared to *Xcc* wild type (Table S2, and Fig. 7D). Interestingly, the major differences between the two strains within the unsaturated fatty acid (UFA) were in the branched UFAs (BUFAs): the percentage of non‐branched UFA was similar in the wild type and the mutant, the percentage of iso BUFAs was significantly higher in the mutant, and the percentage of anteiso BUFAs was slightly higher in the wild type (Table S2, and Fig. 7D). These data shows that TfmR has a significant effect on the fatty acid abundance, potentially due to its effect on the expression of fatty acid catabolism genes.

### Long‐chain fatty acids suppress T3SS gene expression

**Figure 8:**
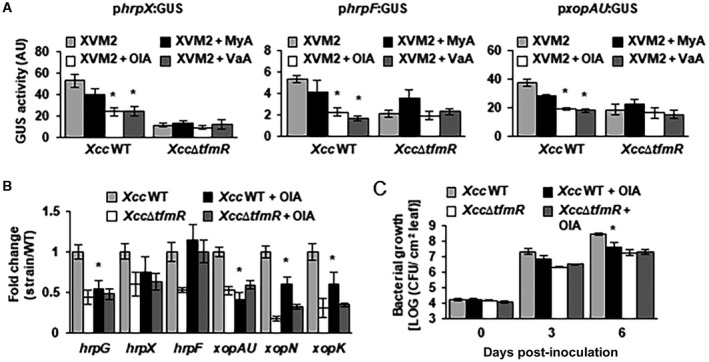
Long‐chain fatty acids suppress T3SS and T3E gene expression. (A) GUS activity was determined for *Xcc* wild type (WT) and *Xcc*∆*tfmR* harbouring the indicated promoter fusions. Bacteria were grown for 24  h in XVM2 media supplemented with the indicated fatty acids. Values are means ± SE of at least three independent biological repeats within one experiment. The experiments were repeated three times with similar results. (B) mRNA abundance of the indicated genes was quantified by qRT‐PCR in bacterial cultures after incubation in XVM2 or XVM2 + 0.1% oleic acid (OlA) for 24 h. *gyrA* mRNA abundance was used for normalization. Values are means ± SE of five independent biological repeats within one experiment. The experiment was repeated twice with similar results. (C) Bacterial growth *in planta*. Prior to inoculation bacteria were incubated in XVM2 or XVM2 supplemented with 0.1% oleic acid (OlA) for 24 h. Values are means ± SE of five or six independent biological repeats within one experiment. The experiments were repeated two (for *Xcc*∆*tfmR*) or three (for *Xcc* wild type) times with similar results. (A, B, C) Asterisks indicate a significant difference (Student’s *t*‐test, *P‐*value < 0.05) compared to bacteria, which were incubated in XVM2 without fatty acids.

We demonstrated that TfmR functions as a suppressor of fatty acid catabolism genes in the absence of long‐chain fatty acids; therefore we hypothesized that fatty acid sensing or metabolism plays a role in T3SS gene expression. We tested the induction of the *phrpX:GUS*, *phrpF*:GUS and *pxopAU:GUS *in *Xcc* wild type and *Xcc∆tfmR *incubated in XVM2 media supplemented with 0.1% of myristic acid, oleic acid and vaccenic acid. The addition of oleic acid and vaccenic acid significantly (*P*‐value < 0.05) reduced the expression of the three reporters in *Xcc* wild type when compared to XVM2 media without fatty acids (Fig. 8A). The induction of the three reporters was not altered in *Xcc∆tfmR *by the addition of fatty acids (Fig. 8A). We further tested the effect of oleic acid on T3SS gene expression by quantification of T3SS and effector genes (e.g. *hrpG, hrpX, hrpF, xopAU, xopN* and *xopK*) using qRT‐PCR. In the wild‐type *Xcc*, expression of the majority of the marker genes was reduced in XVM2 supplemented with 0.1% oleic acid compared to XVM2 (Fig. 8B). We did not observe a similar effect in *Xcc∆tfmR *(Fig. 8B).

The effect of oleic acid on the virulence of *Xcc* was also examined. We first attempted to add oleic acid to the inoculation medium and directly infiltrated the bacteria with oleic acid into sweet orange leaves, but we failed to observe any difference in virulence. We then examined the effect of oleic acid on the bacteria by pre‐incubating them with XVM2 or XVM2 with 0.1% oleic acid for 24 h prior to leaf infiltration. *Xcc* that were pre‐incubated with oleic acid displayed consistent retardation in growth compared to the control incubation in *Xcc* wild type but not in* Xcc∆tfmR *(Fig. 8C). These results indicate that fatty acids or derivatives of fatty acid metabolism play an essential role in the regulation of the T3SS and virulence in *Xcc* and suggest that TfmR acts as a fatty acid sensor that modulates this response.

## Discussion

In this study we identified the TetR‐family transcriptional repressor TfmR as a long‐chain fatty acid sensor and virulence regulator in *Xanthomonas*. We showed that deletion of *tfmR* in *Xcc* resulted in reduction of type III secretion and effector gene expression and subsequently attenuated the pathogenicity of *Xcc* *in planta*. Further analysis discovered that TfmR directly represses the expression of fatty acid metabolism associated genes and that its DNA binding ability is disrupted by long‐chain fatty acids. In turn, we demonstrated that long‐chain fatty acids attenuate the expression of type III secretion and effector gene in wild‐type *Xcc* but not in the *tfmR* mutant; revealing a novel link between fatty acid sensing and virulence regulation in *Xcc.*

In most pathogenic bacteria virulence gene expression is under multilayer metabolic control mediated by numerous inputs (Olive and Sassetti, 2016; Rohmer *et al*., 2011). The HrpG/HrpX virulence regulon is well characterized in *Xanthomonas* but the specific environmental cues that control this regulon are not well understood. In all *Xanthomonas* spp. the *hrpG/hrpX* regulon is suppressed in complex rich media and induced in an optimized defined media (Astua‐Monge *et al*., 2005; Jiang *et al*., 2013; Schulte, 1992; Tsuge *et al*., 2002). This effect suggests that metabolic regulation of this regulon is likely to be partially under negative control by specific metabolites. In support of this hypothesis, we identified that supplementing long‐chain fatty acids to the *Xcc* T3SS inducing media, XVM2, suppressed the expression of *hrpG*, *hrpX* and their downstream T3SS genes. In the plant apoplast simple carbohydrates are available for the bacteria to utilize as a carbon source (Lowell *et al*., 1989) (Fatima and Senthil‐Kumar, 2015; Naseem *et al*., 2017) while long‐chain fatty acids are usually associated with non‐specific lipid transfer proteins (nsLTPs) making them less accessible (Liu *et al*., 2015). On the other hand, plant leaf and fruit surface areas are enriched in hydroxylated C16 and C18 fatty acids that compose the cutin polymer, the main structural component of the plant cuticle (Lara *et al*., 2015). Previous study of temporal and spatial gene expression in the phyllosphere has shown that the expression of *hrpG* is repressed when bacteria are localized on the leaf surface and induced once the bacteria reaches the stomata area and enter the plant apoplast (Zhang *et al*., 2009). Therefore the negative effect of long‐chain fatty acids on HrpG/HrpX virulence regulon might function as a form of metabolic environmental sensing by distinguishing the apoplast from the outer leaf surface.

Fatty acids play an essential role in the membrane, energy metabolism and signalling and their biosynthesis and degradation are subjected to tight regulation (Fujita *et al*., 2007). The regulation of Fad in Enterobacteria is mediated by the global GntR‐family transcription regulator FadR (Fujita *et al*., 2007; Simons *et al*., 1980). While the beta‐oxidation pathway is conserved in *Xanthomonas*, it does not encode a homolog of FadR. TfmR binds to its target promoters and is released from them in the presence of long‐chain fatty acid ligand. We found that this regulator represses the expression of *fadE* and *fadH* genes while not affecting the expression of the downstream beta‐oxidation coding *fadB* and *fadA* genes in the experimental conditions that were tested. FadE catalyzes the production of enoyl‐CoA from acyl‐CoA and FadH functions as an accessory enzyme that recues 2, 4‐dienoyl‐CoA to enoyl‐CoA when the initial FadE substrate is an UFA with double bonds (Campbell and Cronan, 2002; He *et al*., 1997). We speculate that TfmR functions as a regulator of enoyl‐CoA production while other compartments of the beta‐oxidation pathway are regulated separately. Interestingly, the *Xanthomonas* *fadB* and *fadA* are encoded in an operon along with a TetR‐family gene coding gene (XAC2014), which shares homology (36% identity, 54% positive) to *Pseudomonas aeruginosa* PsrA. PsrA was implicated to regulate the *fadB/fadA* operon in *Pseudomonas *(Kang *et al*., 2008); suggesting that in *Xanthomonas* the beta‐oxidation pathway is controlled at least by two independent regulators.

TfmR might contribute to virulence by affecting multiple independent pathways. While we show that expression of T3SS and effector marker genes is reduced in *Xcc*∆*tfmR*, the overexpression of HrpG only partially salvages the virulence of *Xcc∆tfmR *indicating that other factors contribute to the reduction in virulence. Indeed, *Xcc∆tfmR *displays multiple phenotypes that can affect in planta fitness independent of the T3SS. For example, reduced production of EPS and altered membrane fatty acid composition will potentially make the bacteria more sensitive to plant antimicrobial compounds (Katzen *et al*., 1998; Li and Wang, 2011a; Ramos *et al*., 2002). Furthermore, inability to utilize sucrose efficiently as a carbon source will hinder the bacterial growth during infection since sucrose is abundant in the plant and is speculated to serve as one of the main carbon sources utilized by the bacteria (Fatima and Senthil‐Kumar, 2015).

The mechanism by which TfmR and long‐chain fatty acids regulate the *hrpG/hrpX* regulon remains elusive. Direct transcriptional control of the regulon by TfmR is unlikely since TfmR does not bind to the *hrpG* and *hrpX* promoter regions. We speculated that the *hrpG/hrpX* regulon might be indirectly affected by the constitutive expression of *mhpC, fadE* or *fadH.* However, we disproved this hypothesis in this study. The HrpG and HrpX were found to be directly controlled in multiple ways and several regulators were identified to affect these proteins in different levels (Andrade *et al*., 2014; Li *et al*., 2014; Lu *et al*., 2011; Zhou *et al*., 2018); transcriptional control of one or several of these regulators by TfmR is possible. Alternatively, the effect of TfmR and long‐chain fatty acids on the *hrpG/hrpX* regulon might be a consequence of altered cell metabolic state. Another mechanism that might play a role in the function of TfmR is production and response to DSF (Zhou *et al*., 2017a). DSF is a medium‐chain fatty acid derivative and its production might be affected by the effect of TfmR on fatty acid metabolism and can also potentially directly affect the ability of TfmR to bind to its target promoters by acting as a fatty acid ligand. To test this hypothesis we monitored the expression of in *rpfB*, *rpfF, rpfC* and *rpfG* in *Xcc∆tfmR *but did not observe any differential gene expression compared to the wild type (Fig. S7). We also tested whether TfmR can bind to the *rpfB*/*rpfF* promoter *in vivo* but did not observe any significant interaction (Fig. S3B). Identification of novel targets of TfmR and further examination of DSF production and sensing by *Xcc∆tfmR* will provide new insights into the effect of TfmR on pathogenicity and cell physiology.

**Figure 9:**
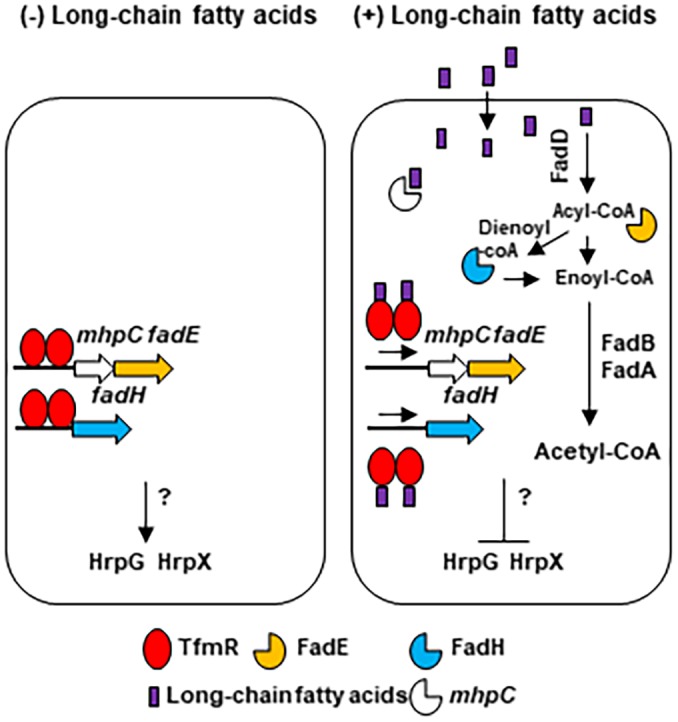
The proposed model of fatty acids mediated regulation of *hrpG*, *hrpX* and beta‐oxidation genes by TfmR. Model represents proposed regulation of *fadE*, *fadH*, *mhpC*, *hrpX* and *hrpG* by TfmR in low abundance of long‐chain fatty acids (left image) and high abundance of long‐chain fatty acids (right image).

While further study should be conducted regarding the link between TfmR and the control of the *hrpG/hrpX* regulon our observation leads to a proposed model (Fig. 9): When bacteria are found in environments that contain low abundance of long‐chain fatty acids, such as the plant apoplast, TfmR binds to the promoter region of *mhpC*, *fadE* and *fadH* to suppress their expression and indirectly promote the expression of the *hrpG/hrpX* regulon. In a long‐chain fatty acid enriched environment, such as the plant leaf surface, long‐chain fatty acids they act a ligand and bind to TfmR thus, releasing it from its target genes and induce the expression of MhpC, FadE and FadH, which allows the utilization of fatty acids as an energy source by introducing them into the beta‐oxidation pathway and produce acetyl‐coA. In parallel, binding of long chain‐fatty acids by TfmR suppress the expression of the *hrpG/hrpX* regulon through an unknown mechanism. By catabolizing the fatty acids through the beta‐oxidation pathway the level of long‐chain fatty acids is reduced, freeing TfmR and allowing it to again bind to and suppress the expression of its target genes and keeping lipid homeostasis.

## Experimental Procedures

### Bacterial strains, plasmids and primers

The bacterial strains and plasmids used in this study are listed in Table S3. Oligonucleotides used in this study are listed in Table S4. *Xanthomonas citri* was grown at 28 °C in NB medium (Beef extract 3 g/L, Peptone 5 g/L), nutrient agar (NA) plates, XVM2 media (Wengelnik and Bonas, 1996) or modified XVM2 media (XVM) in which fructose and sucrose were replaced with 0.1% oleic acid. *E. coli* was grown at 28 °C in Luria‐Bertani (LB) medium at 37 °C. When required, growth media was supplemented with gentamicin (5 μg/mL), kanamycin (50 μg/mL), ampicillin (100 μg/mL) and spectinomycin (200 μg/mL).

### Tn5 transposon screen

*Xcc* 306 bacteria containing pGUS:p*hrpF *reporter were transformed with Tn5 transposon using EZ‐Tn5™ <KAN‐2>Tnp Transposome™ Kit (Lucigen Corporation, Middleton, WI, USA) and plated on XVM2 agar (1.5%) supplemented with X‐gluc (0.01 mg/mL), gentamicin and kanamycin yielding ~10 000 colonies. Five hundred colonies, which displayed reduced or enhanced intensity of blue compared to neighbouring colonies were picked into new XVM2/X‐gluc plates in parallel to *Xcc* 306 + pGUS:p*hrpF*. Two hundred and forty colonies that displayed consistent difference in GUS activity from *Xcc* 306 were used for plant inoculation assays on Valencia sweet orange and symptoms were scored visually 5 days after inoculation. The transposon integration site was determined in 16 clones, which displayed reduced virulence compared to *Xcc* 306 in three independent experimental repeats using touchdown PCR (Levano‐Garcia *et al*., 2005).

### Production of *Xcc* deletion and complemented strains

To produce deletion mutants in *tfmR*, *mhpC* and *fadE*, 740 bp to 900 bp fragments (according to primers listed in Table S4) upstream and downstream of the ORF were amplified with genomic DNA of *Xcc* 306. The two amplicons were combined together into one fragment using overlap PCR and cloned into the pOK1 suicide vector (Huguet *et al*., 1998). The deletion vector was transformed into *Xcc* by electroporation and markerless deletion mutants were produced using a two‐step sucrose counter‐selection procedure (Zhou *et al*., 2015). For complementation of *Xcc*∆*tfmR*, TfmR (XAC3052) or *hrpG* (XAC1265) were amplified from genomic DNA of *Xcc* 306, cloned into pBBR1MCS‐5 or pBBR1MCS‐2 (Kovach *et al*., 1995) under the control of a *lac* promoter with added fusion of HA‐tag at the C‐terminus. Plasmids were transformed into *Xcc*∆*tfmR*. For overexpression, the operon encoding of *mhpC* and *fadE* (XAC3053 and XAC3054) was amplified from *Xcc* 306, cloned into pBBR1MCS‐5 and introduced into *Xcc* wild type.

### Motility, EPS production, extracellular enzymes and catalase activity and stress resistance assays

Motility and production of exopolysaccharides (EPS) were quantified as previously described (Li and Wang, 2011b). For analysis of protease, amylase and cellulase activity bacteria (10^8 ^CFU/mL) were spotted on NA plates containing 1% skimmed milk, 1% carboxymethyl cellulose or 0.5% starch and extracellular activity was quantified as described previously (Szczesny *et al*., 2010; Zhou *et al*., 2017b). Catalase activity assay was conducted as described by (Zhou *et al*., 2017b); H_2_O_2 _degradation was quantified using Pierce Quantitative Peroxide Assay Kit (Thermo Fisher scientific, Waltham, MA, USA). Resistance to SDS and H_2_O_2_ were conducted as described by (Li and Wang, 2012).

### Pathogenicity and HR assays

For pathogenicity assays expanded leaves of 2‐year‐old Valencia sweet orange were infiltrated with bacterial suspensions (10^6^ CFU/mL for monitoring bacterial growth; 10^8^ CFU/mL for monitoring symptoms development) in 10 mM MgCl_2_ using a needless syringe and plants were kept in a greenhouse at 28 °C under natural light conditions. Bacterial growth was quantified as previously described (Teper *et al*., 2018).

For monitoring HR‐like cell death and quantification of ion leakage 4‐week‐old *N.* *benthamiana* plants were infiltrated with 5 x 10^8^ CFU/mL *Xcc* bacteria in 10 mM MgCl_2_ using a needless syringe. Ion leakage was measured as described (Teper *et al*., 2018) using CON 700 benchtop conductivity meter (OAKTON Instruments, Vernon Hills, Il, USA).

### GUS assays

To generate GUS reporter plasmid (pGUS) *E. coli* β‐Glucuronidase (*gus*) gene followed by a T7 terminator were cloned in reverse orientation to the *lac* promoter in pBBR1MCS‐5. To generate GUS promoter fusions constructs the putative promoter regions of the tested genes (elaborated in Table S3) was cloned upstream to the *gus* gene in pGUS.

GUS assay was conducted as described (Jiang *et al*., 2009). GUS activity was quantified by arbitrary units (AU) and determined as A405 / (time in min × total protein in µg × 0.02).

### RNA isolation and qRT‐PCR

RNA was isolated from bacteria and plant tissue using TRIzol™ Reagent (Invitrogen, Carlsbad, CA, USA) according to provided instructions. RNA samples (1 μg) were reverse‐transcribed using qScript cDNA Synthesis Kit (Quanta BioSciences, Inc. Gaithersburg MD, USA) and subjected to qRT‐PCR using gene specific primers (Table S4). cDNAs were amplified using the SYBR Premix Ex Taq II (Clontech Laboratories, Inc. Mountain View, CA, USA) and the QuantStudio 3 Real‐Time PCR System (Applied Biosystems Inc., Foster City, CA). The *Xcc gyrA* and citrus *GAPDH* genes were used for normalization and gene expression was calculated by the comparative *Ct *method (Pfaffl, 2001).

### Protein purification and electrophoretic mobility shift assay

*TfmR* was cloned into the pGEX‐4T‐1 GST fusion expression vector and transformed into *E. coli* Rosetta strain. GST‐TfmR was purified as previously described (Teper *et al*., 2018).

Probe oligonucleotides were amplified from genomic DNA of *Xcc* 306 using 5′ Biotin‐TEG modified primers (IDT Inc, Coralville, IA, USA) (Table S4). EMSA was conducted using the LightShift Chemiluminescent EMSA Kit (Thermo Fisher, Waltham, MA, USA) according to manufacturer’s instructions. For determination of DNA binding affinity band intensity was quantified using ImageJ (https://imagej.nih.gov/ij/) and KD value was calculated as previously described (Heffler *et al*., 2012).

### Chromatin Immunoprecipitation‐Quantitative Polymerase Chain Reaction (ChIP‐qPCR)

ChIP assays were done as described by (Pandey *et al*., 2016) with modifications. Briefly, 100 mL of *Xcc*∆*tfmR* contacting pBBR1MCS‐5 (empty vector control) or pBBR1MCS‐5:*tfmR‐HA* were grown for 36 h in NB media. Cells were pelleted, and re‐suspended in Tris‐buffered saline (TBS) and cross‐linked with 0.75% formaldehyde for 30 min. Fixed cells were washed with TBS and sonicated until DNA was broken into 500 bp to 1000 bp fragments. For immunoprecipitation, cell lysate was incubated with Monoclonal Anti‐HA−Agarose (Sigma‐Aldrich, St. Louis, MO, USA) as and IP was performed as instructed by the manufacturer. Protein‐DNA complex was eluted as described (Pandey *et al*., 2016). Relative representation of potential target promoter sequences was quantified using qPCR (primers listed in Table S4) samples were normalized by using primers directed to the *gyrA* promoter, which was found to not interact with TfmR *in vitro*.

### Fatty acid methyl ester analysis

Three‐day old cultures of *Xcc* wild type and *Xcc∆tfmR *were recultured on three NA plates and incubated at 28 °C for 48 h and 50 mg of bacteria were scooped from each NA plate and frozen in liquid nitrogen. Samples were saponificated, methylated and analysed for fatty acid content using gas chromatography by Microbial ID, Inc. (Newark, DE, USA).

## Supporting information


**Fig. S1** GUS activity and virulence analysis of *Xcc* Tn5 transposon mutants. (A) Bacterial cultures of *Xcc* wild type and the indicated Tn5 *Xcc* mutants carrying the p*hrpF*:GUS reporter plasmid were streaked on XVM2 X‐gluc plates and incubated at 28 °C. Plates were photographed 4 days later. (B) GUS activity in liquid XVM2 media was quantified in *Xcc* wild type and the indicated Tn5 *Xcc* mutants carrying the p*hrpF*:GUS reporter plasmid using PNPG as a substrate. GUS activity values were standardized to a value representing relative activity to *Xcc* wild type. Values are means ± SE of six independent biological repeats taken from three separate experiments (each containing two repeats). Asterisks indicate a significant difference (Student’s *t*‐test, *P*‐value < 0.05) relative to *Xcc* wild type. (C) Sweet orange leaves were syringe‐infiltrated with suspensions (1 × 10^8^ CFU) of *Xcc* wild type (WT) and the indicated Tn5 *Xcc* mutants. Plants were photographed 5 days later. The experiments were repeated at least three times with similar results.

**Fig. S2** Characteristics of TfmR. (A) Physical map of the genomic region surrounding XAC3052 *tfmR* showing the transposon insertion sites of both Tn5 mutants, the fragment deleted in *Xcc*Δ*tfmR* (between the two red dotted lines) and the location of the validation primers used in  D  (two black arrows). (B) Sweet orange leaves were syringe‐infiltrated with suspensions (1 × 10^8^ CFU mL) of *Xcc* wild type (WT) and two transposon mutants in *tmfR* (*Xcc* *tfmR*:Tn5‐1 and *Xcc* *tfmR*:Tn5‐2). Photograph was taken at 5 days post‐inoculation. The experiment was repeated three times with similar results. (C) Protein alignment of *E. coli* TetR (PU17_12890) and homologs of TfmR in *Xanthomonadales* using Clustal Omega Multiple Sequence Alignment tool under default setting (https://www.ebi.ec.uk/Tools/msa/clustalo/). The representative TfmR homologs were taken from the genomes of *Xanthomonas citri* subsp. *citri* (*Xcc*, XAC3052), *Xanthomonas albinlineans* GPE PC73R (*Xalb*, XALC_2196), *Stenotrophomonas maltophilia *K279a (*Smlt*, Smlt3646), *Pseudoxanthomonas spadix* BD‐a59 (*Pxs*, DSC_05240), *Lysobacter capsica *55 (*Lbc*, LC55x_1220) and *Luteimonas* sp. JM171 (*Lum*, BGP89_07680). Green marked amino acids indicate the amino acid is completely conserved in all *Xanthomonadales* TfmR homologs, yellow marked amino acids indicate the amino acids are conserved in all *Xanthomonadales* XAC3052 homologs and *E. coli* TetR. (D) Deletion of XAC3052 was validated by PCR using the primers indicated by the black arrows in ‘A’.

**Fig. S3** Characterization of *Xcc*Δ*tfmR*. (A) Mobility assay. Bacterial suspensions (1 × 10^8^ CFU/mL) of the indicated strains were spotted (5 µL) on soft NA plates (0.4%). Colony diameter was quantified 3 days later. Values are means ± SE of three independent biological repeats within one experiment. The experiments were repeated three with similar results. Asterisks indicate a significant difference (Student’s *t*‐test, *P*‐value < 0.05) relative to *Xcc* wild type. (B, C) Extracellular hydrolase activity of *Xcc*Δ*tfmR*. Suspensions (1 × 10^8^ CFU mL) of *Xcc* wild type (WT), *Xcc*Δ*tfmR* and *Xcc*Δ*tfmR *+ *tfmR* were spotted (10 µL) on NA plates containing either 1% carboxymethylcellulose (CMC), 1% skimmed milk or 0.5% starch. Plates were photographed (B) and halo diameter was quantified (C) after incubation period of 72 h. Values are means ± SE of three independent biological repeats within one experiment. The experiments were repeated for two (for starch) or three (for CMC and skimmed milk) with similar results. Asterisks indicate a significant difference (Student’s *t*‐test, *P*‐value < 0.05) relative to *Xcc *wild type. (D) Catalase activity was tested in the cell extracts of the indicated strains. Represented values were standardized for catalase activity of *Xcc* wild type (set as 100%). Values are means ± SE of eight independent biological repeats taken from three separate experiments (each containing two or three repeats). (E) The indicated strains were challenged with 0.03% H_2_O_2_ or 0.1% SDS. Resistance to H_2_O_2_ and SDS was quantified as the relative survival rate of *Xcc*Δ*tfmR* and *Xcc*Δ*tfmR *+ *tfmR* compared to wild type (set as 100). Values are means ± SE of three independent biological repeats within one experiment. The experiments were repeated twice with similar results.

**Fig. S4** Direct interaction between TftR and putative target promoters. (A) EMSA was used to determine the binding of biotin labelled DNA by GST‐TfmR *in vitro*. 35 pmol of GST or 20 pmol of GST‐TfmR was incubated with 2 ng of the indicated DNA fragments. The experiment was repeated four times with similar results. (B) ChIP‐qPCR was used to determine *in vivo* interaction between TftR and the indicated promoters. Fixed extracts from the *Xcc*Δ*tfmR* + *tfmR*‐*HA* or *Xcc*Δ*tfmR* + empty vector (EV) were fragmented and the immunoprecipitated (IP) using HA agarose. Representation of the promoter sequences was standardized to the relative representation of the *gyrA* promoter. Values represent the average relative representation of the indicated promoter sequences in six independent (conducted in three separated experiments) TfmR HA IP samples compared to EV control after logarithmic transformation. Asterisks indicate a significant difference (Student’s *t*‐test, *P*‐value < 0.05) compared to the EV.

**Fig. S5** The contribution of TfmR to virulence is not dependent on *mhpC* *fadE*. Sweet orange leaves were syringe‐infiltrated with suspensions (1 × 10^8^ CFU/mL for A and C, 1 × 10^6^ CFU/mL for B and D) of the indicated *Xcc* strains. (A, C) pictures were taken 5 days post‐inoculation. The experiments were repeated three times with similar results. (B, D) *In planta* bacterial population was determined at the indicated days post inoculation. Values are means ± SE of at least three independent biological repeats within one experiment. The experiments were repeated twice with similar results. Letters denote significant differences based on analysis of variance (ANOVA) and comparisons for all pairs using Student’s *t*‐test (*P*‐value < 0.05).

**Fig. S6** Regulation of beta oxidation pathway genes by TfmR. (A, B) Growth of *Xcc* wild type (WT), *Xcc*Δ*tfmR*, *Xcc*Δ*tfmR* + *tfmR* in NB media (A) or NB media containing 0.1% oleic acid (B). Values are means ± SE of three independent biological repeats within one experiment. The experiments were repeated three times with similar results. (C) GIS activity in bacterial cultures grown in NB or XVM2 media was determined for *Xcc* wild type (WT) and *Xcc*Δ*tfmR* harbouring the indicated GUS promoter fusions. Values are means ± SE of three independent biological repeats within one experiment. The experiments were repeated three times with similar results. Asterisks indicate a significant difference (Student’s *t*‐test, *P*‐value < 0.05) relative to *Xcc* wild type. (D) EMSA was used to determine the binding of 1 ng of biotin labelled DNA representing the indicated promoters by 35 nmol of GST or 20 pmol of GST‐TfmR. The experiment was repeated twice with similar results. (E) Inhibition or promoter binding by TfmR by addition of 100 ng competitor DNA (X100 competitor). The experiment was repeated twice with similar results.

**Fig. S7** mRNA abundance of the indicated genes was quantified by qRT‐PCR in bacterial cultures after incubation in XVM2 media for 24 h. *gyrA* mRNA abundance was used for normalization. Values are means ± SE of three independent biological repeats within one experiment. The experiments were repeated at least three times with similar results. No significant difference was observed (Student’s *t*‐test, *P*‐value < 0.05) between the *Xcc* wild type (WT) and *Xcc*Δ*tfmR*.

**Table S1** Virulence deficient Tn5 mutants identified in this study.

**Table S2** Fatty acid abundance (%) in *Xcc* wild type and *Xcc*∆*tfmR.*

**Table S3** Bacterial strains and plasmids used in this study.

**Table S4** Primers used during this study.
