# Supplementary figures and images for: TfmR, a novel TetR‐family transcriptional regulator, modulates the virulence of Xanthomonas citri in response to fatty acids
Source: Mol Plant Pathol. 2019 Mar 27;20(5):701–15. doi: 10.1111/mpp.12786 (PMC6637906; doi:10.1111/mpp.12786)

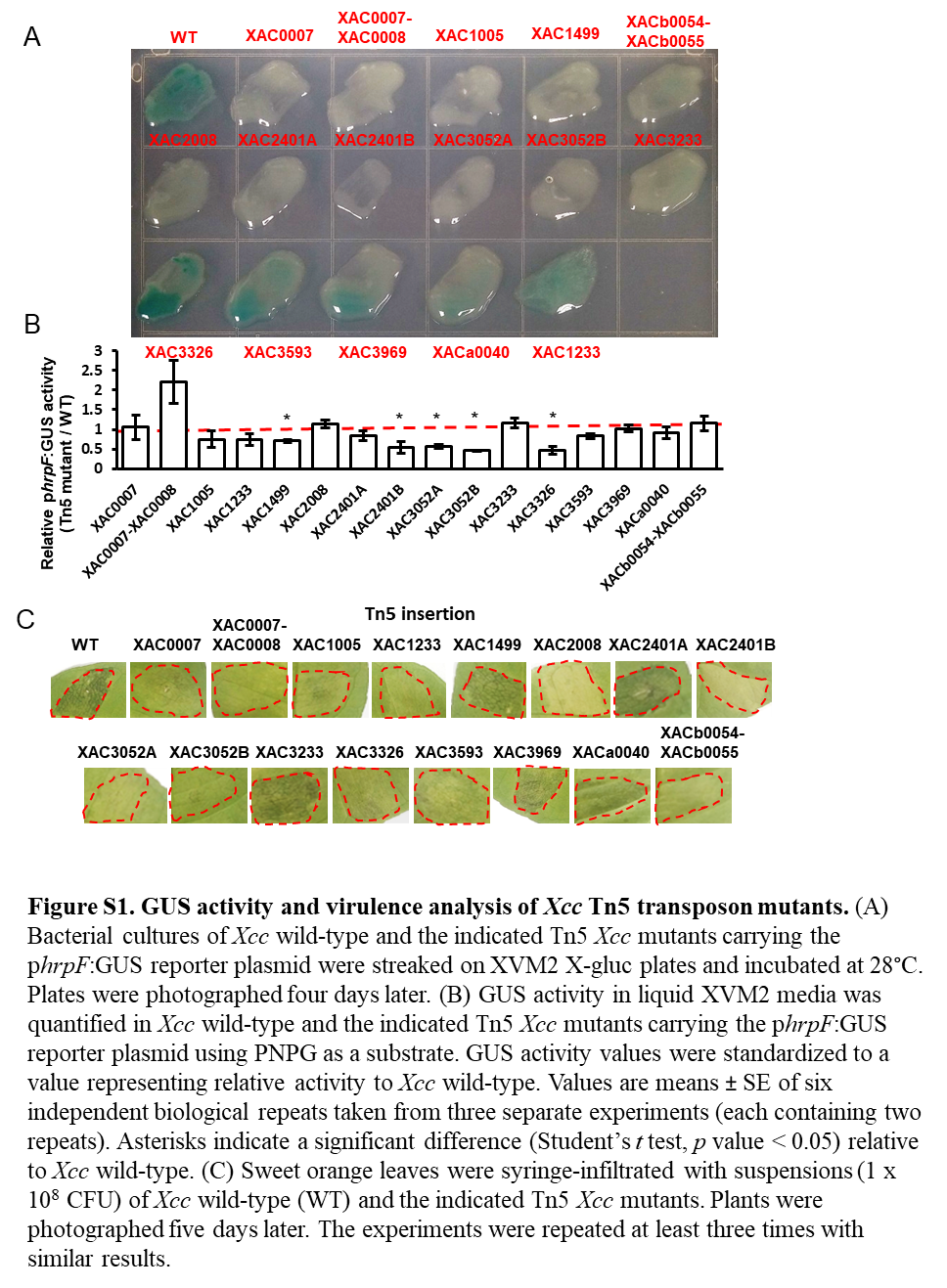

Supplement: Supplementary file 1 — Fig. S1 GUS activity and virulence analysis of Xcc Tn5 transposon mutants. (A) Bacterial cultures of Xcc wild type and the indicated Tn5 Xcc mutants carrying the phrpF:GUS reporter plasmid were streaked on XVM2 X‐gluc plates and incubated at 28 °C. Plates were photographed 4 days later. (B) GUS activity in liquid XVM2 media was quantified in Xcc wild type and the indicated Tn5 Xcc mutants carrying the phrpF:GUS reporter plasmid using PNPG as a substrate. GUS activity values were standardized to a value representing relative activity to Xcc wild type. Values are means ± SE of six independent biological repeats taken from three separate experiments (each containing two repeats). Asterisks indicate a significant difference (Student’s t‐test, P‐value < 0.05) relative to Xcc wild type. (C) Sweet orange leaves were syringe‐infiltrated with suspensions (1 × 10^8^ CFU) of Xcc wild type (WT) and the indicated Tn5 Xcc mutants. Plants were photographed 5 days later. The experiments were repeated at least three times with similar results. [file MPP-20-701-s001.docx]

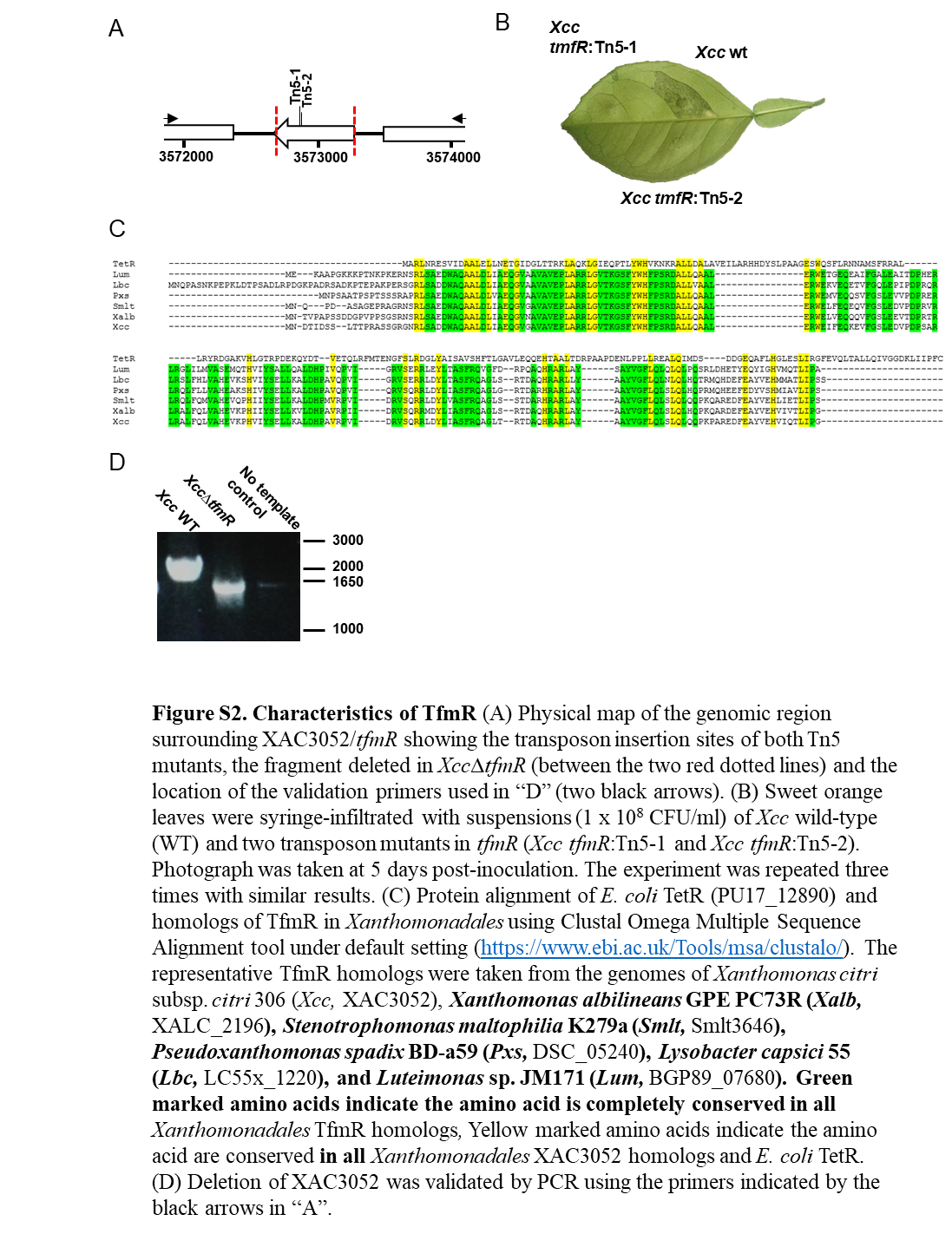

Supplement: Supplementary file 2 — Fig. S2 Characteristics of TfmR. (A) Physical map of the genomic region surrounding XAC3052 tfmR showing the transposon insertion sites of both Tn5 mutants, the fragment deleted in XccΔtfmR (between the two red dotted lines) and the location of the validation primers used in D (two black arrows). (B) Sweet orange leaves were syringe‐infiltrated with suspensions (1 × 10^8^ CFU mL) of Xcc wild type (WT) and two transposon mutants in tmfR (Xcc tfmR:Tn5‐1 and Xcc tfmR:Tn5‐2). Photograph was taken at 5 days post‐inoculation. The experiment was repeated three times with similar results. (C) Protein alignment of E. coli TetR (PU17_12890) and homologs of TfmR in Xanthomonadales using Clustal Omega Multiple Sequence Alignment tool under default setting (https://www.ebi.ec.uk/Tools/msa/clustalo/). The representative TfmR homologs were taken from the genomes of Xanthomonas citri subsp. citri (Xcc, XAC3052), Xanthomonas albinlineans GPE PC73R (Xalb, XALC_2196), Stenotrophomonas maltophilia K279a (Smlt, Smlt3646), Pseudoxanthomonas spadix BD‐a59 (Pxs, DSC_05240), Lysobacter capsica 55 (Lbc, LC55x_1220) and Luteimonas sp. JM171 (Lum, BGP89_07680). Green marked amino acids indicate the amino acid is completely conserved in all Xanthomonadales TfmR homologs, yellow marked amino acids indicate the amino acids are conserved in all Xanthomonadales XAC3052 homologs and E. coli TetR. (D) Deletion of XAC3052 was validated by PCR using the primers indicated by the black arrows in ‘A’. [file MPP-20-701-s002.docx]

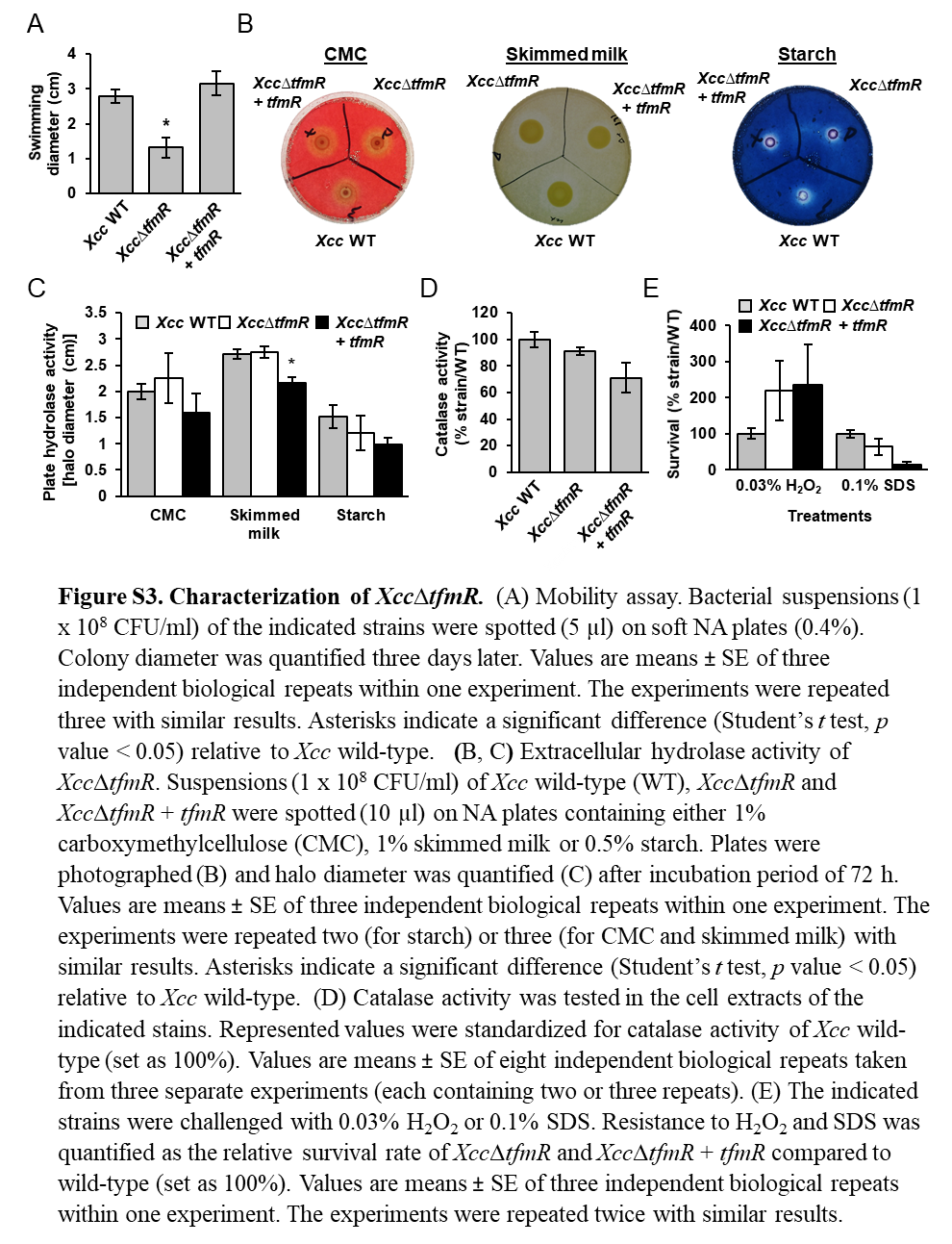

Supplement: Supplementary file 3 — Fig. S3 Characterization of XccΔtfmR. (A) Mobility assay. Bacterial suspensions (1 × 10^8^ CFU/mL) of the indicated strains were spotted (5 µL) on soft NA plates (0.4%). Colony diameter was quantified 3 days later. Values are means ± SE of three independent biological repeats within one experiment. The experiments were repeated three with similar results. Asterisks indicate a significant difference (Student’s t‐test, P‐value < 0.05) relative to Xcc wild type. (B, C) Extracellular hydrolase activity of XccΔtfmR. Suspensions (1 × 10^8^ CFU mL) of Xcc wild type (WT), XccΔtfmR and XccΔtfmR + tfmR were spotted (10 µL) on NA plates containing either 1% carboxymethylcellulose (CMC), 1% skimmed milk or 0.5% starch. Plates were photographed (B) and halo diameter was quantified (C) after incubation period of 72 h. Values are means ± SE of three independent biological repeats within one experiment. The experiments were repeated for two (for starch) or three (for CMC and skimmed milk) with similar results. Asterisks indicate a significant difference (Student’s t‐test, P‐value < 0.05) relative to Xcc wild type. (D) Catalase activity was tested in the cell extracts of the indicated strains. Represented values were standardized for catalase activity of Xcc wild type (set as 100%). Values are means ± SE of eight independent biological repeats taken from three separate experiments (each containing two or three repeats). (E) The indicated strains were challenged with 0.03% H2O2 or 0.1% SDS. Resistance to H2O2 and SDS was quantified as the relative survival rate of XccΔtfmR and XccΔtfmR + tfmR compared to wild type (set as 100). Values are means ± SE of three independent biological repeats within one experiment. The experiments were repeated twice with similar results. [file MPP-20-701-s003.docx]

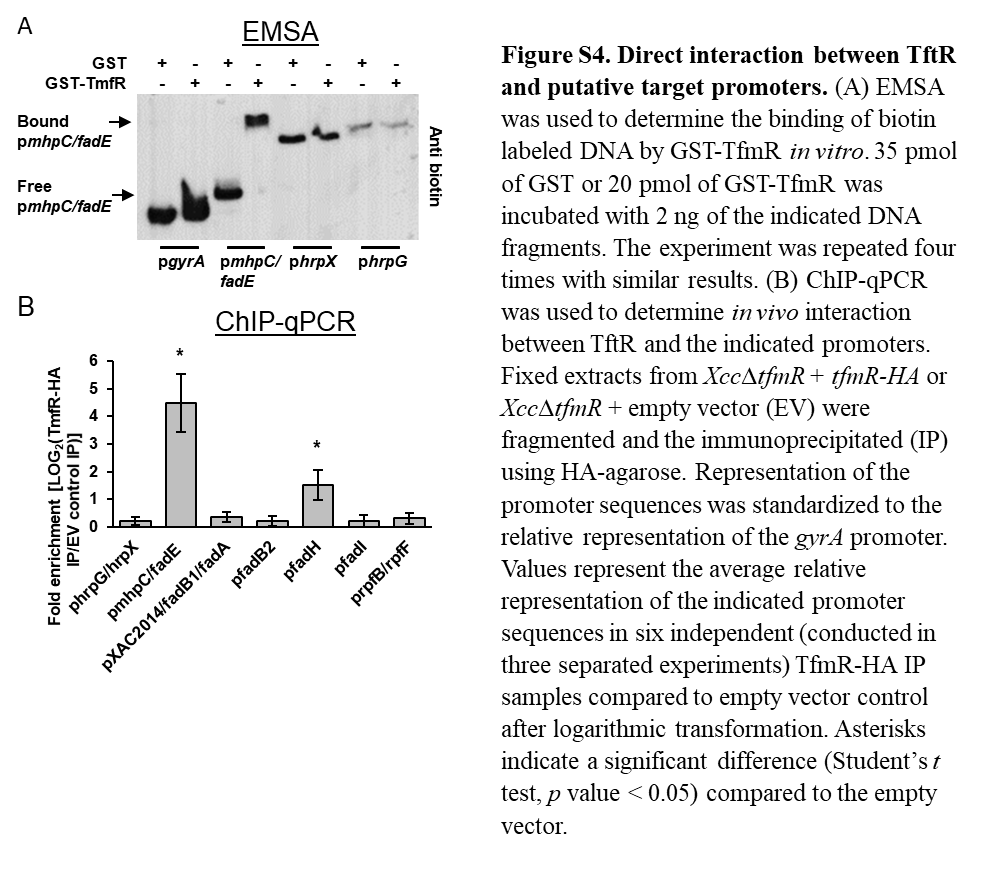

Supplement: Supplementary file 4 — Fig. S4 Direct interaction between TftR and putative target promoters. (A) EMSA was used to determine the binding of biotin labelled DNA by GST‐TfmR in vitro. 35 pmol of GST or 20 pmol of GST‐TfmR was incubated with 2 ng of the indicated DNA fragments. The experiment was repeated four times with similar results. (B) ChIP‐qPCR was used to determine in vivo interaction between TftR and the indicated promoters. Fixed extracts from the XccΔtfmR + tfmR‐HA or XccΔtfmR + empty vector (EV) were fragmented and the immunoprecipitated (IP) using HA agarose. Representation of the promoter sequences was standardized to the relative representation of the gyrA promoter. Values represent the average relative representation of the indicated promoter sequences in six independent (conducted in three separated experiments) TfmR HA IP samples compared to EV control after logarithmic transformation. Asterisks indicate a significant difference (Student’s t‐test, P‐value < 0.05) compared to the EV. [file MPP-20-701-s004.docx]

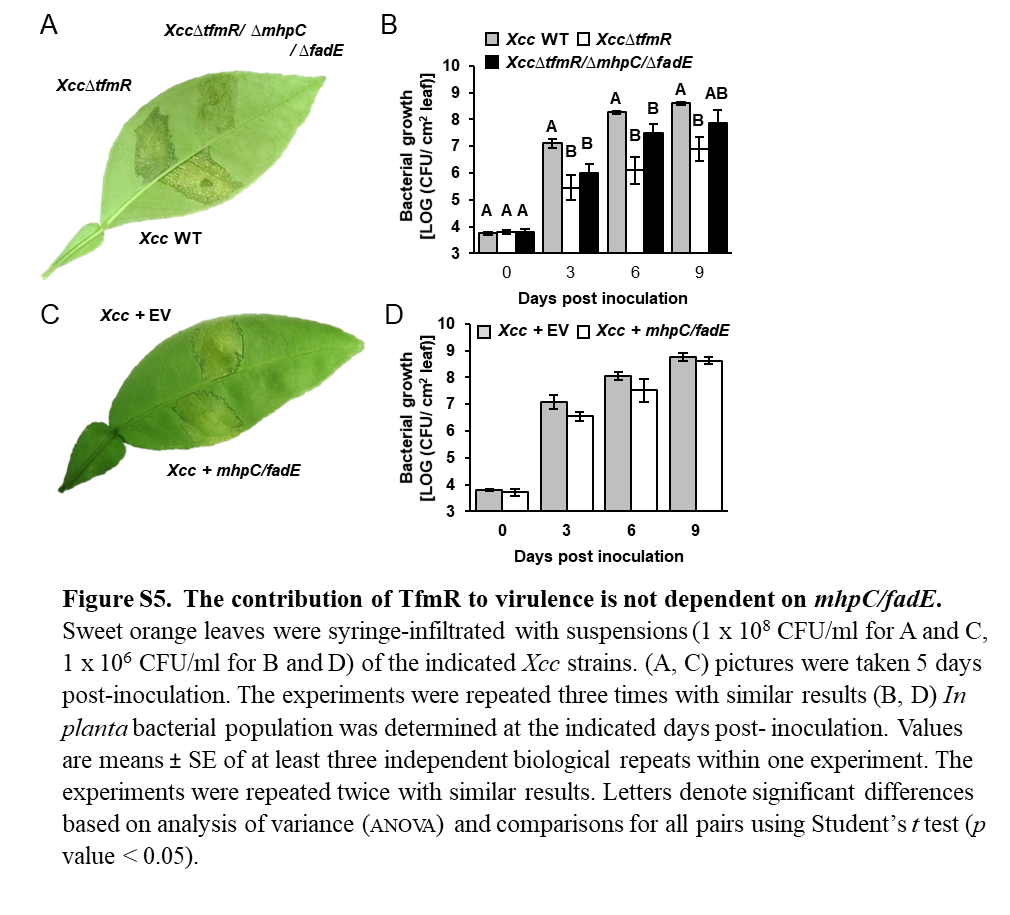

Supplement: Supplementary file 5 — Fig. S5 The contribution of TfmR to virulence is not dependent on mhpC fadE. Sweet orange leaves were syringe‐infiltrated with suspensions (1 × 10^8^ CFU/mL for A and C, 1 × 10^6^ CFU/mL for B and D) of the indicated Xcc strains. (A, C) pictures were taken 5 days post‐inoculation. The experiments were repeated three times with similar results. (B, D) In planta bacterial population was determined at the indicated days post inoculation. Values are means ± SE of at least three independent biological repeats within one experiment. The experiments were repeated twice with similar results. Letters denote significant differences based on analysis of variance (ANOVA) and comparisons for all pairs using Student’s t‐test (P‐value < 0.05). [file MPP-20-701-s005.docx]

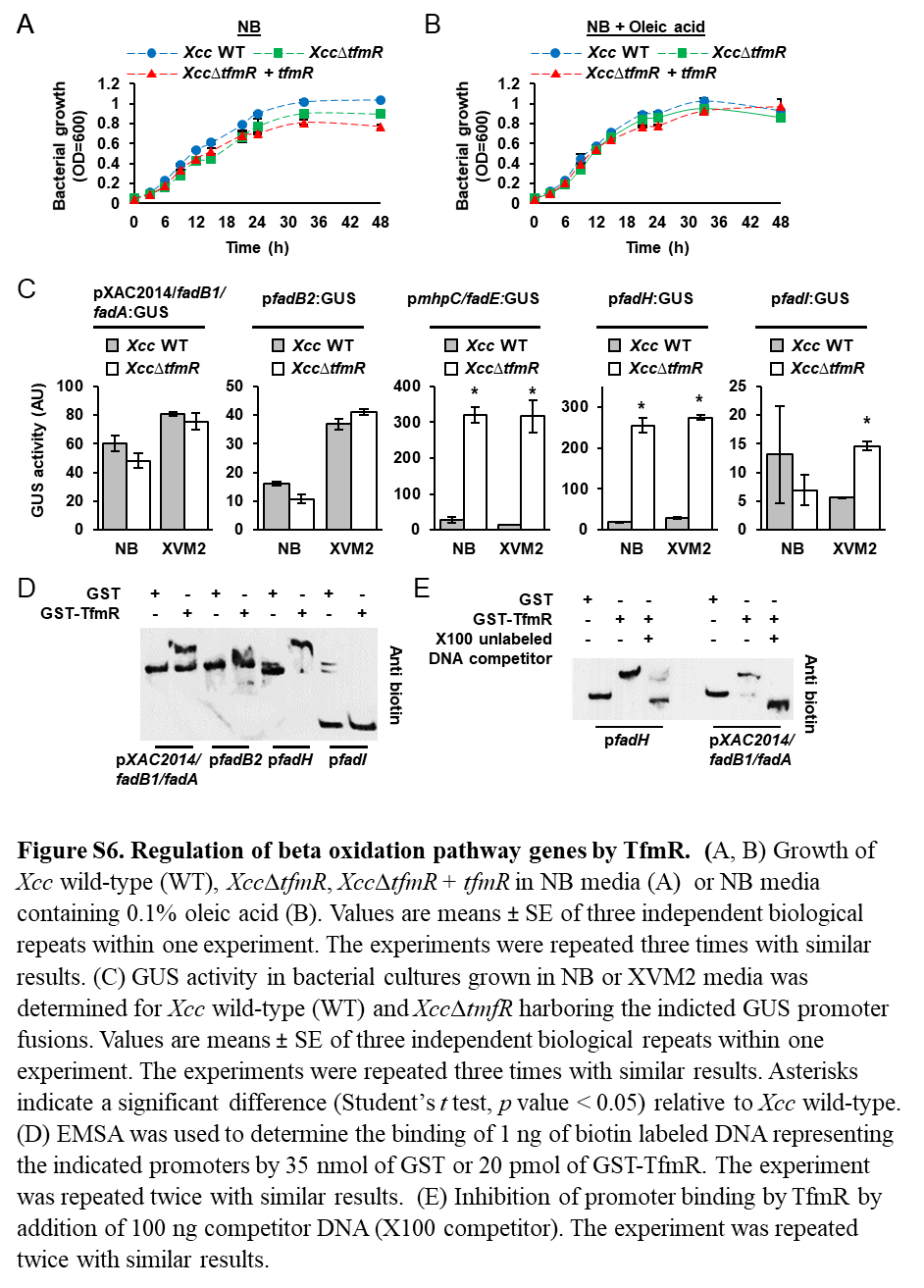

Supplement: Supplementary file 6 — Fig. S6 Regulation of beta oxidation pathway genes by TfmR. (A, B) Growth of Xcc wild type (WT), XccΔtfmR, XccΔtfmR + tfmR in NB media (A) or NB media containing 0.1% oleic acid (B). Values are means ± SE of three independent biological repeats within one experiment. The experiments were repeated three times with similar results. (C) GIS activity in bacterial cultures grown in NB or XVM2 media was determined for Xcc wild type (WT) and XccΔtfmR harbouring the indicated GUS promoter fusions. Values are means ± SE of three independent biological repeats within one experiment. The experiments were repeated three times with similar results. Asterisks indicate a significant difference (Student’s t‐test, P‐value < 0.05) relative to Xcc wild type. (D) EMSA was used to determine the binding of 1 ng of biotin labelled DNA representing the indicated promoters by 35 nmol of GST or 20 pmol of GST‐TfmR. The experiment was repeated twice with similar results. (E) Inhibition or promoter binding by TfmR by addition of 100 ng competitor DNA (X100 competitor). The experiment was repeated twice with similar results. [file MPP-20-701-s006.docx]

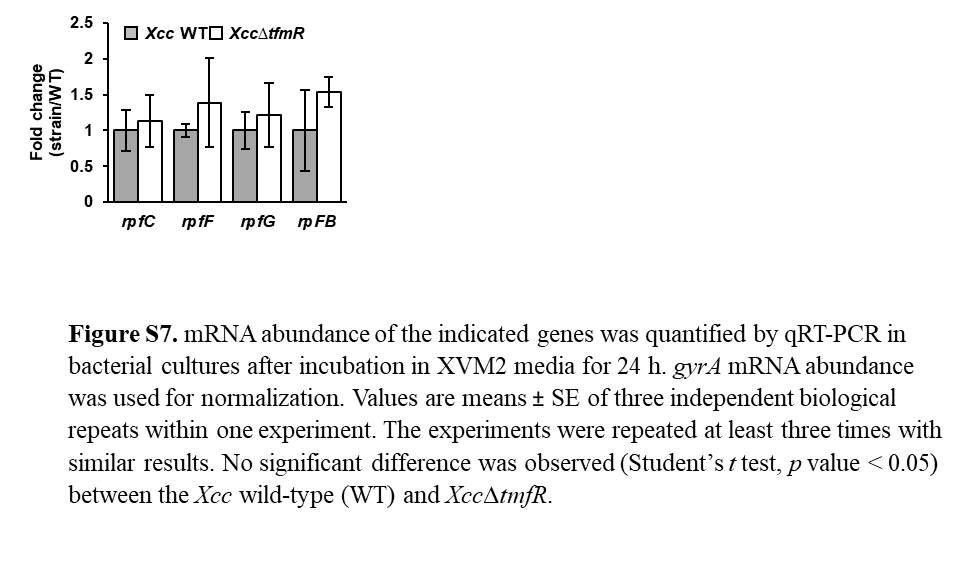

Supplement: Supplementary file 7 — Fig. S7 mRNA abundance of the indicated genes was quantified by qRT‐PCR in bacterial cultures after incubation in XVM2 media for 24 h. gyrA mRNA abundance was used for normalization. Values are means ± SE of three independent biological repeats within one experiment. The experiments were repeated at least three times with similar results. No significant difference was observed (Student’s t‐test, P‐value < 0.05) between the Xcc wild type (WT) and XccΔtfmR. [file MPP-20-701-s007.docx]
