# Supplementary material for: TfmR, a novel TetR‐family transcriptional regulator, modulates the virulence of Xanthomonas citri in response to fatty acids
Source: Mol Plant Pathol. 2019 Mar 27;20(5):701–15. doi: 10.1111/mpp.12786 (PMC6637906; doi:10.1111/mpp.12786)
Supplement: Supplementary file 8 — Table S1 Virulence deficient Tn5 mutants identified in this study. [file MPP-20-701-s008.docx]

**Table S1:** Virulence deficient Tn5 mutants identified in this study

| Gene ID | Function of gene product | Insertion site^1^ | p*hrpF*:GUS expression - XVM2 plate^2^ | p*hrpF*:GUS expression - XVM2 liquid media^3^ | Virulence^4^ |
| --- | --- | --- | --- | --- | --- |
|  | Wild-type *Xcc* 306 |  | +++++ | +++++ | +++++ |
| XAC0007 | Transmembrane protein, tpr repeats | 10448^G^ | + | ++++++ | - |
| XAC0007-XAC0008 | Intergenic region | 10894^G^ | ++ | +++++ | - |
| XAC1005 | peptidyl-prolyl cis-trans isomerase | 1160626^G^ | ++ | ++ | +++ |
| XAC1233 | D-alanyl-D-alanine carboxypeptidase | 1411112^G^ | ++++++ | +++ | + |
| XAC1499 | Transcriptional regulator, helix-turn-helix XRE-family like | 1733639^G^ | + | +++++ | ++++ |
| XAC2008 | Outer membrane lipoprotein carrier protein LolA | 2346457^G^ | ++ | +++++ | +++ |
| XAC2401 | Acetoacetyl-CoA reductase | 2797451^G^ | ++ | +++ | ++++ |
| XAC2401 | Acetoacetyl-CoA reductase | 2797929^G^ | + | ++ | - |
| XAC3052 | Transcriptional regulator, tetR family | 3572791^G^ | + | ++ | ++ |
| XAC3052 | Transcriptional regulator, tetR family | 3572807^G^ | + | ++ | ++ |
| XAC3233 | Transposase | 3809506^G^ | + | +++++ | ++++ |
| XAC3326 | Cation/multidrug efflux pump | 3916031^G^ | ++++++ | ++ | + |
| XAC3593 | NAD dependent epimerase/dehydratase/dehydrogenase | 4261365^G^ | ++++++ | ++++ | +++ |
| XAC3969 | Unknown | 4664410^G^ | + | +++++ | ++++ |
| XACa0040 | Unknown | 31893^A^ | +++ | +++++ | +++ |
| XACb0054-XACb0055 | Intergenic region | 47838^B^ | +++ | +++++ | - |

1. G indicates the Tn5 insertion site was in the core genome of *Xcc* 306 (NC_003919.1), A indicates Tn5 insertion site was in the pXAC33 plasmid (NC_003921.3), B indicates Tn5 insertion site was in the pXAC64 plasmid (NC_003922).
2. p*hrpF*:GUS expression in the plate was scored visually in colonies gown in XVM2 plates with 0.01 mg/ml X-gluc according to the intensity of blue. Color range was scored from “-“ which represents no color change to “++++++” which represents the strongest intensity. Wild-type *Xcc* harboring the p*hrpF*:GUS reporter was used as a reference (top raw). The experiment was repeated three times with similar result.
3. p*hrpF*:GUS expression in the liquid media was scored according to three independent quantitative GUS activity assays from bacteria grown in liquid XVM2 culture. Bacteria were grown for 12 h in XVM2, cells were lysed and GUS activity was determined using p-Nitrophenyl-β-D-glucuronide. Wild-type *Xcc* harboring the p*hrpF*:GUS reporter was used as a reference (top raw).
4. Virulence examined in sweet orange leaves which were syringe-infiltrated with suspensions (1 x 10^8^ CFU/ml) of *Xcc*. Virulence was visually scored at 5 days post inoculation and ranged from “-“ which represents no canker symptoms to “+++++” which represents strong canker symptoms equivalent to Wild-type *Xcc*. The experiment was repeated three times with similar result.
