# Supplementary material for: TfmR, a novel TetR‐family transcriptional regulator, modulates the virulence of Xanthomonas citri in response to fatty acids
Source: Mol Plant Pathol. 2019 Mar 27;20(5):701–15. doi: 10.1111/mpp.12786 (PMC6637906; doi:10.1111/mpp.12786)
Supplement: Supplementary file 9 — Table S2 Fatty acid abundance (%) in Xcc wild type and Xcc∆tfmR. [file MPP-20-701-s009.docx]

**Table S2:** Fatty acid abundance (%) in *Xcc* wild-type and *Xcc*∆*tfmR*

| **Fatty acid** | ***Xcc* wild-type** | | | ***Xcc*∆*tfmR*** | | |
| --- | --- | --- | --- | --- | --- | --- |
|  | Repeat 1 | Repeat 2 | Repeat 3 | Repeat 1 | Repeat 2 | Repeat 3 |
| 10:0 | 0.39 | 0.44 | 0.41 | 0.54 | 0.50 | 0.53 |
| 11:0 iso | 2.09 | 2.31 | 2.07 | 3.01 | 3.04 | 3.06 |
| 11:0 anteiso | 0.13 | 0.12 | 0.14 | 0 | 0 | 0 |
| 10:0 2OH | 0.15 | 0.14 | 0 | 0 | 0 | 0 |
| 10:0 3OH | 0.24 | 0.21 | 0.14 | 0.19 | 0.19 | 0.20 |
| 12:1 at 11-12 | 0.12 | 0.14 | 0.13 | 0 | 0 | 0 |
| 11:0 iso 3OH | 1.53 | 1.52 | 1.61 | 1.54 | 1.52 | 1.55 |
| 11:0 3OH | 0.50 | 0.45 | 0.56 | 0.27 | 0.24 | 0.26 |
| 13:0 iso | 0.46 | 0.48 | 0.49 | 3.16 | 3.28 | 3.98 |
| 13:0 anteiso | 0.09 | 0.11 | 0.12 | 0.58 | 0.59 | 0.68 |
| 12:0 iso 3OH | 0.39 | 0.35 | 0.41 | 0.35 | 0.43 | 0.35 |
| 12:0 3OH | 2.60 | 2.64 | 2.67 | 2.73 | 2.63 | 2.76 |
| 14:0 iso | 0.74 | 0.75 | 0.77 | 2.05 | 2.15 | 2.24 |
| 14:1 w5c | 0.17 | 0.21 | 0.18 | 1.10 | 1.16 | 1.30 |
| 14:0 | 1.06 | 1.04 | 1.09 | 2.71 | 2.76 | 3.00 |
| 13:0 iso 3OH | 3.78 | 3.80 | 3.91 | 3.82 | 3.77 | 4.02 |
| 13:0 2OH | 0.38 | 0.38 | 0.41 | 0.22 | 0.19 | 0.22 |
| 15:1 iso F | 0.23 | 0.27 | 0.25 | 0.16 | 0.16 | 0.20 |
| 13:0 3OH | 0.22 | 0.26 | 0.23 | 0.43 | 0.43 | 0.49 |
| 15:0 iso | 28.14 | 28.00 | 28.17 | 40.25 | 41.03 | 41.15 |
| 15:0 anteiso | 13.56 | 13.13 | 13.59 | 9.36 | 8.69 | 8.05 |
| 15:1 w8c | 0.10 | 0.14 | 0.10 | 0 | 0 | 0 |
| 15:1 w6c | 1.27 | 1.56 | 1.26 | 1.66 | 1.63 | 1.79 |
| 15:0 | 2.16 | 1.66 | 2.17 | 0.78 | 0.76 | 0.79 |
| 16:0 iso | 3.37 | 3.35 | 3.34 | 2.33 | 2.51 | 2.04 |
| 16:1 w7c | 17.21 | 17.78 | 17.30 | 13.15 | 12.87 | 12.85 |
| 16:1 w5c | 0.09 | 0.15 | 0 | 0.22 | 0.22 | 0.25 |
| 16:0 | 3.44 | 2.90 | 3.38 | 1.73 | 1.71 | 1.53 |
| 15:0 iso 3OH | 0 | 0 | 0 | 0.21 | 0.21 | 0.24 |
| 15:0 2OH | 0 | 0 | 0 | 0.12 | 0.12 | 0 |
| 17:1 iso w9c | 5.54 | 5.70 | 5.55 | 3.17 | 3.20 | 2.76 |
| 17:1 anteiso B | 0 | 0 | 0 | 0 | 0 | 0.27 |
| 17:1 iso I | 0.30 | 0.34 | 0.27 | 0.28 | 0.27 | 0 |
| 17:0 iso | 4.96 | 5.02 | 4.86 | 2.24 | 2.30 | 1.80 |
| 17:0 anteiso | 0.65 | 0.66 | 0.62 | 0.23 | 0.21 | 0.18 |
| 17:1 w8c | 1.78 | 1.77 | 1.72 | 0.41 | 0.40 | 0.37 |
| 17:1 w6c | 0.45 | 0.53 | 0.39 | 0.27 | 0.26 | 0.26 |
| 17:0 | 0.33 | 0.23 | 0.25 | 0 | 0 | 0 |
| 16:0 3OH | 0.10 | 0.12 | 0 | 0.23 | 0.15 | 0.25 |
| 17:0 iso 3OH | 0.28 | 0.29 | 0.27 | 0 | 0 | 0 |
| 18:1 w9c | 0.65 | 0.69 | 0.69 | 0.38 | 0.29 | 0.43 |
| 18:1 w7c | 0.35 | 0.38 | 0.35 | 0.15 | 0 | 0 |
| **Saturated fatty acids** | **28.25651** | **29.64298** | **28.19522** | **20.45699** | **20.47647** | **20.62758** |
| **Unbranched unsaturated fatty acids** | **11.56418** | **10.46439** | **11.3131** | **9.695419** | **10.03304** | **9.888074** |
| **Branched unsaturated fatty acids with *iso* conformation** | **45.74374** | **45.8733** | **45.89516** | **60.24742** | **60.44024** | **59.87948** |
| **Branched unsaturated fatty acids with *anteiso* conformation** | **14.43556** | **14.01934** | **14.464** | **9.483333** | **9.176334** | **9.607947** |
