# Supplementary material for: TfmR, a novel TetR‐family transcriptional regulator, modulates the virulence of Xanthomonas citri in response to fatty acids
Source: Mol Plant Pathol. 2019 Mar 27;20(5):701–15. doi: 10.1111/mpp.12786 (PMC6637906; doi:10.1111/mpp.12786)
Supplement: Supplementary file 10 — Table S3 Bacterial strains and plasmids used in this study. [file MPP-20-701-s010.docx]

**Table S3**: Bacterial strains and plasmids used in this study

| Strain or plasmid | Relevant characteristic | Reference |
| --- | --- | --- |
| **Strain** | | |
| ***Escherichia coli*** | | |
| HST08 | F–, endA1, supE44, thi-1, recA1, relA1, gyrA96, phoA, Φ80d lacZΔ M15, Δ (lacZYA - argF) U169, Δ (mrr - hsdRMS - mcrBC), ΔmcrA, λ– | Clontech Laboratories Inc, Mountain View, CA |
| S-17-1 λpir | RK2 tra regulon, pir, host for pir-dependent plasmid pOK1 | (1) |
| Rosetta | BL21 derivative, for expression of fusion proteins | MERCK, Kenilworth  NJ, USA |
| ***Xanthomonas citri* subsp *citri*** | | |
| 306 | Wild-type | (2) |
| *Xcc*∆*tfmR* | XAC3052 deletion strain in the background of *Xcc* 306 | This study |
| *Xcc* *tmfR*:Tn5-1 | Tn5 insertion mutant in XAC3052 in the background of *Xcc* 306, Kn^R*^ | This study |
| *Xcc* *tmfR:*Tn5-2 | Tn5 insertion mutant in XAC3052 in the background of *Xcc* 306, Kn^R^ | This study |
| *Xcc* ∆*tfmR*∆*mhpC*∆*fadE* | XAC3052/XAC3053/XAC3054 deletion strain in the background of *Xcc*∆*tfmR* | This study |
| *Xcc hrcV*:Tn5 | Tn5 insertion mutant in XAC0405 in the background of *Xcc* 306, Kn^R^ | (3) |
| *Xcc* XAC0007:Tn5 | Tn5 insertion mutant in XAC0007 in the background of *Xcc* 306, Kn^R^ | This study |
| *Xcc* XAC0007-XAC0008:Tn5 | Tn5 insertion mutant in intergenic region between XAC0007 to XAC0008 in the background of *Xcc* 306, Kn^R^ | This study |
| *Xcc* XAC1005:Tn5 | Tn5 insertion mutant in XAC1005 in the background of *Xcc* 306, Kn^R^ | This study |
| *Xcc* XAC1499:Tn5 | Tn5 insertion mutant in XAC1449 in the background of *Xcc* 306, Kn^R^ | This study |
| *Xcc* XAC1233:Tn5 | Tn5 insertion mutant in XAC1233 in the background of *Xcc* 306, Kn^R^ | This study |
| *Xcc* XAC2008:Tn5 | Tn5 insertion mutant in XAC2008 in the background of *Xcc* 306, Kn^R^ | This study |
| *Xcc* XAC2401:Tn5-1 | Tn5 insertion mutant in XAC2401 in the background of *Xcc* 306, Kn^R^ | This study |
| *Xcc* XAC2402:Tn5-2 | Tn5 insertion mutant in XAC2401 in the background of *Xcc* 306, Kn^R^ | This study |
| *Xcc* XAC3233:Tn5 | Tn5 insertion mutant in XAC3233 in the background of *Xcc* 306, Kn^R^ | This study |
| *Xcc* XAC3326:Tn5 | Tn5 insertion mutant in XAC3326 in the background of *Xcc* 306, Kn^R^ | This study |
| *Xcc* XAC3593:Tn5 | Tn5 insertion mutant in XAC3593 in the background of *Xcc* 306, Kn^R^ | This study |
| *Xcc* XAC3969:Tn5 | Tn5 insertion mutant in XAC3969 in the background of *Xcc* 306, Kn^R^ | This study |
| *Xcc* XACa0040:Tn5 | Tn5 insertion mutant in XACa0040 in the background of *Xcc* 306, Kn^R^ | This study |
| *Xcc* XACb0054-XACb0055:Tn5 | Tn5 insertion mutant in intergenic region between XACb0054 to XACb0055 in the background of *Xcc* 306, Kn^R^ | This study |
| **Plasmid** | | |
| pBBR1MCS-2 | Broad host expression vector. Kn^R^ | (4) |
| pBBR1MCS-2:*hrpG* | pBBR1MCS-2 derivative for expression of *hrpG* fused to HA tag. Kn^R^ | This study |
| pBBR1MCS-5 | Broad host expression vector. Gn^R^ | (4) |
| pBBR1MCS-5:*tfmR* | pBBR1MCS-5 derivative for expression of XAC3052 fused to HA-tag. Gn^R^ | This study |
| pBBR1MCS-5: *mhpC*/*fadE* | pBBR1MCS-5 derivative for expression of the XAC3053/XAC3054 operon. Gn^R^ | This study |
| pGUS | pBBR1MCS-5 derivative containing *gus* gene followed by T7 terminator cloned in reverse orientation of lac promoter . | This study |
| pGUS p*gyrA* | pGUS derivative. The 189 bp upstream region of XAC1631 was cloned upstream to *gus*. Gn^R^ | This study |
| pGUS p*hrpX* | pGUS derivative. The 474 bp upstream region of XAC1266 was cloned upstream to *gus*. Gn^R^ | This study |
| pGUS p*hrpF* | pGUS derivative. The 831 bp upstream region of XAC0394 was cloned upstream to *gus*. Gn^R^ | This study |
| pGUS p*xopAU* | pGUS derivative. The 1000 bp upstream region of XAC1171 was cloned upstream to *gus*. Gn^R^ | This study |
| pGUS p*mhpC*/*fadE* | pGUS derivative. The 217 bp upstream region of the XAC3053/XAC3054 operon was cloned upstream to *gus*. Gn^R^ | This study |
| pGUS pXAC3052 | pGUS derivative. The 217 bp upstream region of XAC3052 was cloned upstream to *gus*. Gn^R^ | This study |
| pGUS p*fadB1/fadA* | pGUS derivative. The 218 bp upstream region of the XAC2014/XAC2013/XAC2012 operon was cloned upstream to *gus*. Gn^R^ | This study |
| pGUS p*fadB2* | pGUS derivative. The 235 bp upstream region of XAC1318 was cloned upstream to *gus*. Gn^R^ | This study |
| pGUS p*fadH* | pGUS derivative. The 206 bp upstream region of XAC1010 was cloned upstream to *gus*. Gn^R^ | This study |
| pGUS p*fadI* | pGUS derivative. The 68 bp upstream region of XAC0213 was cloned upstream to *gus*. Gn^R^ | This study |
| pOK1 | *sacB* *sacQ* *mobRK2* *oriR6K*, Suicide vector. Sp^R^ | (5) |
| pOK1*tfmR* | pOK1 derivative contacting the 741 bp 5’ and 879 bp 3’ flanking regions of XAC3052. Sp^R^ | This study |
| pOK1 *tfmR/mhpC*/*fadE* | pOK1 derivative contacting the 814 bp 5’ and 868 bp 3’ flanking regions of the genomic area encoding for XAC3052, XAC3053 and XAC3054. Sp^R^ | This study |
| pGEX-4T-1 | GST-fusion expression vector. Ap^R^ | GE healthcare, Little Chalfont, UK |
| pGEX-4T-1:XAC3052 | For expression of GST-XAC3052 in *E. coli*. Ap^R^ | This study |

*Kn^R^, Gn^R^ , Sp^R^ and Ap^R^ indicate resistance to kanamycin, gentamicin, spectinomycinand ampicillin, respectively.

**References:**

1. Simon R, Priefer U, Pühler A. 1983. A broad host range mobilization system for in vivo genetic engineering: Transposon mutagenesis in gram negative bacteria. Bio/Technology 1:784–791.

2. da Silva a CR, Ferro J a, Reinach FC, Farah CS, Furlan LR, Quaggio RB, Monteiro-Vitorello CB, Van Sluys M a, Almeida NF, Alves LMC, do Amaral a M, Bertolini MC, Camargo LE a, Camarotte G, Cannavan F, Cardozo J, Chambergo F, Ciapina LP, Cicarelli RMB, Coutinho LL, Cursino-Santos JR, El-Dorry H, Faria JB, Ferreira a JS, Ferreira RCC, Ferro MIT, Formighieri EF, Franco MC, Greggio CC, Gruber a, Katsuyama a M, Kishi LT, Leite RP, Lemos EGM, Lemos MVF, Locali EC, Machado M a, Madeira a MBN, Martinez-Rossi NM, Martins EC, Meidanis J, Menck CFM, Miyaki CY, Moon DH, Moreira LM, Novo MTM, Okura VK, Oliveira MC, Oliveira VR, Pereira H a, Rossi a, Sena J a D, Silva C, de Souza RF, Spinola L a F, Takita M a, Tamura RE, Teixeira EC, Tezza RID, Trindade dos Santos M, Truffi D, Tsai SM, White FF, Setubal JC, Kitajima JP. 2002. Comparison of the genomes of two Xanthomonas pathogens with differing host specificities. Nature 417:459–463.

3. Yan Q, Wang N. 2012. High-throughput screening and analysis of genes of Xanthomonas citri subsp. citri involved in citrus canker symptom development. Mol Plant Microbe Interact 25:69–84.

4. Kovach ME, Elzer PH, Steven Hill D, Robertson GT, Farris MA, Roop RM, Peterson KM. 1995. Four new derivatives of the broad-host-range cloning vector pBBR1MCS, carrying different antibiotic-resistance cassettes. Gene 166:175–176.

5. Huguet E, Hahn K, Wengelnik K, Bonas U. 1998. hpaA mutants of Xanthomonas campestris pv. vesicatoria are affected in pathogenicity but retain the ability to induce host-specific hypersensitive reaction. Mol Microbiol 29:1379–1390.
