# Supplementary material for: TfmR, a novel TetR‐family transcriptional regulator, modulates the virulence of Xanthomonas citri in response to fatty acids
Source: Mol Plant Pathol. 2019 Mar 27;20(5):701–15. doi: 10.1111/mpp.12786 (PMC6637906; doi:10.1111/mpp.12786)
Supplement: Supplementary file 11 — Table S4 Primers used during this study. [file MPP-20-701-s011.docx]

**Table S4**: Primers used during this study

| Primer name | Sequence (5’ to 3’) (Underline represents restriction sites) | Destination vector |
| --- | --- | --- |
| *For genomic deletion* | | |
| 5’flaXAC3052F | CGTGGATCCCAGTGCCAGCGCCAAC | pOK1 |
| 5’flaXAC3052R | CGCTCTTTACAACAACTCTGCTCACATGCCGCGATTTAACCATACGC | pOK1 |
| 3’flaXAC3052F | GCGTATGGTTAAATCGCGGCATGTGAGCAGAGTTGTTGTAAAGAGCG | pOK1 |
| 3’flaXAC3052R | AAAATCTAGAGAACGAGCTGGGAACGCAGACG | pOK1 |
| 5’flaXAC3052/3/4F | AAAATCTAGAAACATCCAAGATGCCTGCAAATACCC | pOK1 |
| 5’flaXAC3052/3/4R | GGTCTATTCTCGCCCCAGCTTATGCCTTATGAGCAGAGTTGTTGTAAAGAGCGTGCTG | pOK1 |
| 3’flaXAC3052/3/4F | CAGCACGCTCTTTACAACAACTCTGCTCATAAGGCATAAGCTGGGGCGAGAATAGACC | pOK1 |
| 3’flaXAC3052/3/4R | CCGGATCCGACAAGCGCGTTTCCGAGTAAGCAC | pOK1 |
| *For complementation* | | |
| CoTfmRORFF | AAAGTCGACGCGTATGGTTAAATCGCGGCATG | pBBR1MCS-5 |
| CoTfmRORFR | CCCCTCTAGACCCCGGAATCAACGTCTGGATC | pBBR1MCS-5 |
| hrpGORFF | CCCAAGCTTCACGCGAAGGCCGCCTAAATG | pBBR1MCS-2 |
| hrpGORFR | AAAGGATCCGCAGGCGGCTGCGTGATG | pBBR1MCS-2 |
| mhpC/fadEF | AAACTCGAGGATGCAGTGCTCGCGGTGTCG | pBBR1MCS-5 |
| mhpC/fadER | CCCCTCTAGACGCGGCTTCGCGTGGCTGTTG | pBBR1MCS-5 |
| *For construction of pGUS* | | |
| GUSF | CCCGAATTCATGTTACGTCCTGTAGAAACCCC | pBBR1MCS-5 |
| GUSR | AAAAAGCTTTCATTGTTTGCCTCCCTGCTG | pBBR1MCS-5 |
| T7TerF | CCCGGTACCCTGAGATCCGGCTGCTAACA | pBBR1MCS-5 |
| T7TerR | AAACTCGAGATCCGGATATAGTTCCTCCT | pBBR1MCS-5 |
| *For GUS reporter assay* | | |
| pgyrAF | AAAGGATCCCTTCCAACGCCGCCCAAAC | pGUS |
| pgyrAR | CCCGAATTCCACGATCACGCTCATGGCGT | pGUS |
| phrpXF | GAGGTCTAGACCAACCTATTGCGATTG | pGUS |
| phrpXR | TTTGAATTCGATCATGCCGGTCTCTCTCTTTG | pGUS |
| phrpFF | GGGGGATCCCAGACCCGCTTCACTGCCG | pGUS |
| phrpFR | TTTGAATTCTGCGCCCATGCGGTGTAGCC | pGUS |
| pxopAUF | CCCGGATCCATGGTCGGCGTCGAATATGG | pGUS |
| pxopAUR | AAAGAATTCGATCTGTTTCATGGTGGAGGCCAG | pGUS |
| pmhpC/fadEF | AAAAGGATCCGCCGCGATTTAACCATACGCTTG | pGUS |
| pmhpC/fadER | CCCCGAATTCCATCGACACCGCGAGCACTGCATC | pGUS |
| ptfmRF | AAAAGGATCCCGACACCGCGAGCACTGCATC | pGUS |
| ptfmRR | CCCCGAATTCCATGCCGCGATTTAACCATACGC | pGUS |
| pfadB1/AF | AAGGATCCGGGAAGTTCTCCGTTGGGCG | pGUS |
| pfadB1/AR | CCGAATTCCATGCGCGAAGACAGGACTCAATC | pGUS |
| pfadB2F | AAGGATCCGTCGGGCCGCACTCCTGTCAG | pGUS |
| pfadB2R | CCGAATTCCATGCGTCTAGCCGTCCGGC | pGUS |
| pfadHF | AAGGATCCGGCGGCGGTCTGGCTCAC | pGUS |
| pfadHR | GGGAATTCGATGTGGATACGCTGGCGTACGG | pGUS |
| pfadIF | AAGGATCCCGGGGTCGAAGCTACACTGCG | pGUS |
| pfadIR | GGGAATTCCATGGGGGACTCGGGGACATG | pGUS |
| *For protein expression* | | |
| ExTfmRORFF | CCCGAATTCATGAACGACACCATCGACTCCAGC | pGEX-4T-1 |
| ExTfmRORFR | AAAGTCGACTCACCCCGGAATCAACGTCTGG | pGEX-4T-1 |
| *For touchdown PCR* | | |
| Tn5out1 | TTGCAGTTTCATTTGATGCTCGATGAG | |
| Tn5out2 | GGCCTCGAGCAAGACGTTTCC | |
| Degenerate1 | CGGAATTCCGTGTTAAATATGGTATTGTGATNGAYKSNGGNTC | |
| Degenerate2 | CGGAATTCCGGATNGAYKSNGGNTC | |
| *For qRT-PCR* | | |
| RTCsGAPDHF | GAAAGGTCTTGCCTGCTTTG | |
| RTCsGAPDHR | TCCTTCTCCAGCCTCACTGT | |
| RTCsLOB1F | TCCACCAACCGAACCATACA | |
| RTCsLOB1R | GGCACTTGCTTCATAGACCAT | |
| RTgyrAF | GTCAAGGAAAAGAAGCTCGAAG | |
| RTgyrAR | GCTGATACAGGTTGTTGAGCAC | |
| RThrpGF | ATCGTGCTTGGACGTTTCGATTGC | |
| RThrpGR | ATTGAAAGGCAGCGCAAGGACTTC | |
| RThrpXF | AGCGATCTCTGCGTTGTCCTAC | |
| RThrpXR | ATACGCATCTTCGGCCTCTTCCTGA | |
| RThrpFF | AAGCAAGCAAAGAACTACACACAG | |
| RThrpFR | GAAGGTATCCTGATTCAGATCGTC | |
| RTxopAUF | GATCCCACTGACACTGTACGAC | |
| RTxopAUR | TGATCATTTGATACTCCTGGAAGA | |
| RTxopNF | AACAACAAGCTCAACCTCCTGTA | |
| RTxopNR | GTAGTTTGTCGGCCTTGATGTC | |
| RTxopKF | ACAGCGCTACCAGTACTTTGTG | |
| RTxopKR | GTACTGGCGGTGTAGGTGATTC | |
| RTmhpCF | GACGACAACACCACGTTTACC | |
| RTmhpCR | CAGTGACATGCTCGGTAGTGTT | |
| RTfadEF | GGGACTTCATCAAGAAGAACAAGT | |
| RTfadER | GAAGACAGCTTCTGGATCACCT | |
| RTfadAF | AAGGTCGCAGAAGAGTGGAAG | |
| RTfadAR | GATTTCGTACGGGCTGATTTC | |
| RTfadB1F | GAGCTCAAAAAGACCTTCGAAAC | |
| RTfadB1R | GTAGCTGTTGAACACCACTTTGTC | |
| RTfadB2F | GTTAACGCATTTTCGCAGGAG | |
| RTfadB2R | TCCTGGAATTCCTTCAGATCG | |
| RTfadHF | GGCTATCTGATCAACGAATTCAC | |
| RTfadHR | CCGGTAGATGATGATGAAGTCC | |
| RTfadIF | TCGATATCTACGAAATCCACGAG | |
| RTfadIR | ATTGATCTTGTCCGGGTCGAT | |
| RTprfFF | CTCCACCATTCGTGTTGATTC | |
| RTprfFR | CCACATTTCTTCCAGCAGTTC | |
| RTprfCF | TGAAAGGAGTGTCCAGCAATC | |
| RTprfCR | CAGTCCGGTCTTCAACTGTTC | |
| RTprfGF | ATGCAGGATGTTTTAGGGAATC | |
| RTprfGR | CCGAAATCGTAGACCTTCAACTC | |
| RTprfBF | CCGTACCACATCTTCGCATT | |
| RTprfBR | TCAGCTCCTTGACGAAACCT | |
| *For EMSA - 5' Biotin-TEG modified primers* | | |
| BiomhpC/fadEF | GCCGCGATTTAACCATACGCTTG | |
| BiopgyrA | CATTCGGGTTCCGTAAGTGAGGTG | |
| BiophrpG | GGGAGAGTGGTCGTTCATTTAGG | |
| BiophrpX | GATCATGCCGGTCTCTCTCTTTG | |
| BiopfadB1/A | GGGAAGTTCTCCGTTGGGCG | |
| BiopfadB2 | GTCGGGCCGCACTCCTGTCAG | |
| BiopfadH | GATGTGGATACGCTGGCGTACGG | |
| BiopfadI | CGG GGT CGA AGC TAC ACT GCG | |
| *For ChIP qPCR* | | |
| pmhpC/fadEF | CATCACGAAATTGACATATCAGC | |
| pmhpC/fadER | CCATACTTAATGGTATGGATGCAC | |
| pfadABRTF | CTTCTTTGCGATGGTCAATTCTAT | |
| pfadABRTR | GAAGACAGGACTCAATCAAACGAT | |
| pfadB2RTF | GAAAAGAAAAGTTCATACCGATCC | |
| pfadB2RTR | GTTATCATCCAGAGGTTGTTTCCT | |
| pfadHRTF | GTGTTACCTACCAGGCGTCTCC | |
| pfadHRTR | GATACGCTGGCGTACGGATT | |
| pfadIRTF | CTTCCGCCGGTCAATTCC | |
| pfadIRTR | ATAATGGCCGCAGTGTAGCTT | |
| prpfBRTF | AGCAAGACCAGCATGCAAA | |
| prpfBRTR | CTACAACAGCTGGCAGCATACC | |
| phrpGRTF | GTACGCAAGTGTGAAACTTTTGGT | |
| pgyrARTF | CCTGCCGTGGTACAATCC | |
| pgyrARTR | CGGGTTCCGTAAGTGAGGT | |
